# Exosome loaded immunomodulatory biomaterials alleviate local immune response in immunocompetent diabetic mice post islet xenotransplantation

**DOI:** 10.1038/s42003-021-02229-4

**Published:** 2021-06-03

**Authors:** M. Rezaa Mohammadi, Samuel Mathew Rodriguez, Jennifer Cam Luong, Shiri Li, Rui Cao, Hamad Alshetaiwi, Hien Lau, Hayk Davtyan, Mathew Blurton Jones, Mahtab Jafari, Kai Kessenbrock, S. Armando Villalta, Paul de Vos, Weian Zhao, Jonathan R. T. Lakey

**Affiliations:** 1 Department of Materials Science and Engineering, University of California Irvine, Irvine, CA USA; 2 Sue and Bill Stem Cell Center, University of California Irvine, Irvine, CA USA; 3 Department of Biomedical Engineering, University of California Irvine, Irvine, CA USA; 4 Department of Surgery, University of California Irvine, Irvine, CA USA; 5 Department of Biological Chemistry, University of California Irvine, Irvine, CA USA; 6 Institute for Memory Impairments and Neurological Disorders, University of California Irvine, Irvine, CA USA; 7 Department of Neurobiology and Behavior, University of California Irvine, Irvine, CA USA; 8 Institute for Immunology, University of California Irvine, Irvine, CA USA; 9 Department of Pharmaceutical Sciences, University of California Irvine, Irvine, CA USA; 10 Department of Pathology and Medical Biology, Section Immunoendocrinology, University of Groningen, University Medical Center Groningen, Groningen, The Netherlands; 11 Chao Family Comprehensive Cancer Center; Edwards Life Sciences Center for Advanced Cardiovascular Technology; Department of Biomedical Engineering, Department of Biological Chemistry, University of California Irvine, Irvine, CA USA

**Keywords:** Biomaterials - cells, Mesenchymal stem cells

## Abstract

Foreign body response (FBR) to biomaterials compromises the function of implants and leads to medical complications. Here, we report a hybrid alginate microcapsule (AlgXO) that attenuated the immune response after implantation, through releasing exosomes derived from human Umbilical Cord Mesenchymal Stem Cells (XOs). Upon release, XOs suppress the local immune microenvironment, where xenotransplantation of rat islets encapsulated in AlgXO led to >170 days euglycemia in immunocompetent mouse model of Type 1 Diabetes. In vitro analyses revealed that XOs suppressed the proliferation of CD3/CD28 activated splenocytes and CD3+ T cells. Comparing suppressive potency of XOs in purified CD3+ T cells versus splenocytes, we found XOs more profoundly suppressed T cells in the splenocytes co-culture, where a heterogenous cell population is present. XOs also suppressed CD3/CD28 activated human peripheral blood mononuclear cells (PBMCs) and reduced their cytokine secretion including IL-2, IL-6, IL-12p70, IL-22, and TNFα. We further demonstrate that XOs mechanism of action is likely mediated via myeloid cells and XOs suppress both murine and human macrophages partly by interfering with NFκB pathway. We propose that through controlled release of XOs, AlgXO provide a promising new platform that could alleviate the local immune response to implantable biomaterials.

## Abstract (web summary)

Mohammadi et al. show that the transplantation of pancreatic islets within mesenchymal-stem cell (MSC) exosome-loaded hybrid alginate microcapsules in diabetic mice lead to diminished immune-based foreign body response and hyperglycemia control. This is due to immunomodulatory behavior of MSC exosomes at the engraftment site where they exert local immunosuppressive effects.

## Introduction

Transplantation of therapeutic cells has been examined as potential treatments for a variety of diseases, including, β-cells replacement therapies^1^, bone marrow transplantation^2^, and Parkinson’s disease^3,4^. Engineerability of cells and their responsiveness to the environmental cues make them living factories that deliver therapeutic agents with appropriate physiological dosing on demand. Yet, the promise of cell-based therapies is hampered by the challenges of achieving safe and effective long-term engraftment. Transplanted cells can elicit a strong immune response, especially if they originate from non-syngeneic sources. Administration of immunosuppressive regimens (i.e., the non-steroidal anti-inflammatory agents) has been proposed to mute such immune responses, however, this approach may lead to detrimental side-effects including hepatocellular, cardiac or renal toxicities^5^, gastrointestinal ulceration, bleeding, and microbial dysbiosis^6,7^.

One notable example of therapeutic cell transplantation is the pancreatic islet transplantation to treat type 1 diabetes (T1D), which has stimulated ~50 years of research and clinical trials. Human trials on islet transplantation initiated with the Edmonton protocol, suggesting >5 years efficacy in some cases^8,9^. However, adverse events through daily administration of immunosuppressive regimen as well as lack of allogeneic cell donors further compromised the clinical practice of Edmonton protocol^7^. Encapsulation of islets within a protective biomaterial has been considered to eliminate this need for chronic immunosuppression^10^. Dating back to the 1980s, islet transplantation within alginate microcapsules was found to prolong the glycemic correction in diabetic rodents^11^. However, limited therapeutic efficacy and transient glycemic control have been reported in follow-up human trials of islet transplantation within alginate microcapsules^10,12–14^. This suggests the restricted functionality of transplants through islet death and/or loss of mass transfer inwards and outwards of microcapsules. The main reasons behind such graft failure are likely to be the islet necrosis (due to the lack of nutrients and oxygen accessibility within the microcapsules)^15^, as well as immune-mediated pericapsular growth and fibrosis^16–20^. The latter is also known as foreign body response (FBR), which creates considerable discomfort for patients and a variety of health complications^21–23^.

Preventing the transplantation-led inflammatory response reduces pericapsular overgrowth and fibrosis. Many studies have demonstrated that islet transplantation within immune-modulator or immune-insulator microcapsules provides long-term euglycemia in immunocompetent diabetic rodents^1,18,24–26^. Various strategies have been employed to modulate and/or mute the local immune response against implants, including the surface-bound immunomodulatory ligands^25^, anti-biofouling surface modification^24,27,28^, and controlled release of anti-inflammatory agents. The controlled-release (or drug eluting) biomaterials could hold and release variety of anti-inflammatory and/or immunomodulatory molecules overtime (e.g., dexamethasone^29^, IL-4^30^, CSF1R inhibitor^18^, and CXCL12^26^). There are two main possible drawbacks with many of these molecular target inhibitors. The first issue is the potential side effects associated with these agents. For instance, CSF1R inhibitors can elicit fatigue/asthenia, edema^31^, and nonreversible grade 3 deafness^32^, and CXCL12 causes toxicity in cerebrocortical neurons^33^. Other molecular targets such as TNFα inhibitors and anti-TGFβ compounds are also linked to a variety of complications in clinical trials^34,35^. The second challenge with molecular inhibitors lies in their inability to regulate a multitude of inflammatory pathways involved in the immune response against biomaterials transplants, including NFκB^36–38^, CSF1R^16,18^, and JAK/STAT^39^ pathways. Thus, it is speculated that the controlled release of agents that regulate multiple inflammatory pathways may better mute the inflammatory response against implants compared to agents that interfere with single targets.

In this context, mesenchymal stromal cells (MSCs, also named as medicinal signaling cells) are recognized to regulate variety of inflammatory pathways including NFκB^40^, JAK/STAT^41^, MyD88^42^, and PI3K/AKT^43^. The current paradigm of MSC treatment is through paracrine factor, which could partly be attributed to MSC-derived exosomes (XOs)^44,45^. While detailed mechanisms behind immunomodulatory effects of XOs are not yet fully understood, they have been recognized for their capability to regulate the function of multiple immune cell types including macrophages^46^, NK cells^47,48^, B cells^49,50^, and T lymphocytes^51^. We thus hypothesized that co-transplantation of XOs within alginate microcapsules (AlgXO) would alleviate the FBR upon implantation. Upon blocking this inflammation, we next hypothesized that transplantation of rat islets within AlgXO would prolong the function of transplanted islets in immunocompetent streptozotocin-induced diabetic mice.

## Results

### Islet xenotransplantation within AlgXO microcapsules delays the graft rejection

We first isolated XOs from umbilical cord-derived MSCs (UC-MSCs) and characterized UC-MSCs and the size, number, and protein biomarkers of their XOs (Supplementary Fig. 1). We chose UC-MSCs due to their availability, non-invasive isolation, rapid proliferation, suitability for scale-up, and superior biological activity^52^. Two types of alginate microcapsules were fabricated, which are regular Ba^2+^ cross-linked ultrapure alginate microcapsules (CTRL) and AlgXO. To fabricate AlgXO, we loaded XOs inside alginate microcapsules (Supplementary Fig. 2a). To quantify XOs within AlgXO, we dissolved microcapsules and collected XOs through ultracentrifugation (Supplementary Fig. 3). Total number of XOs within ~1000 AlgXO was 5.43 × 10^9^ ± 4.84 × 10^9^ (*n* = 4), whereas XOs within CTRL microcapsules were below the detection limit of nanoparticle tracking analysis (NTA; Supplementary Fig. 2b).

**Fig. 1:**
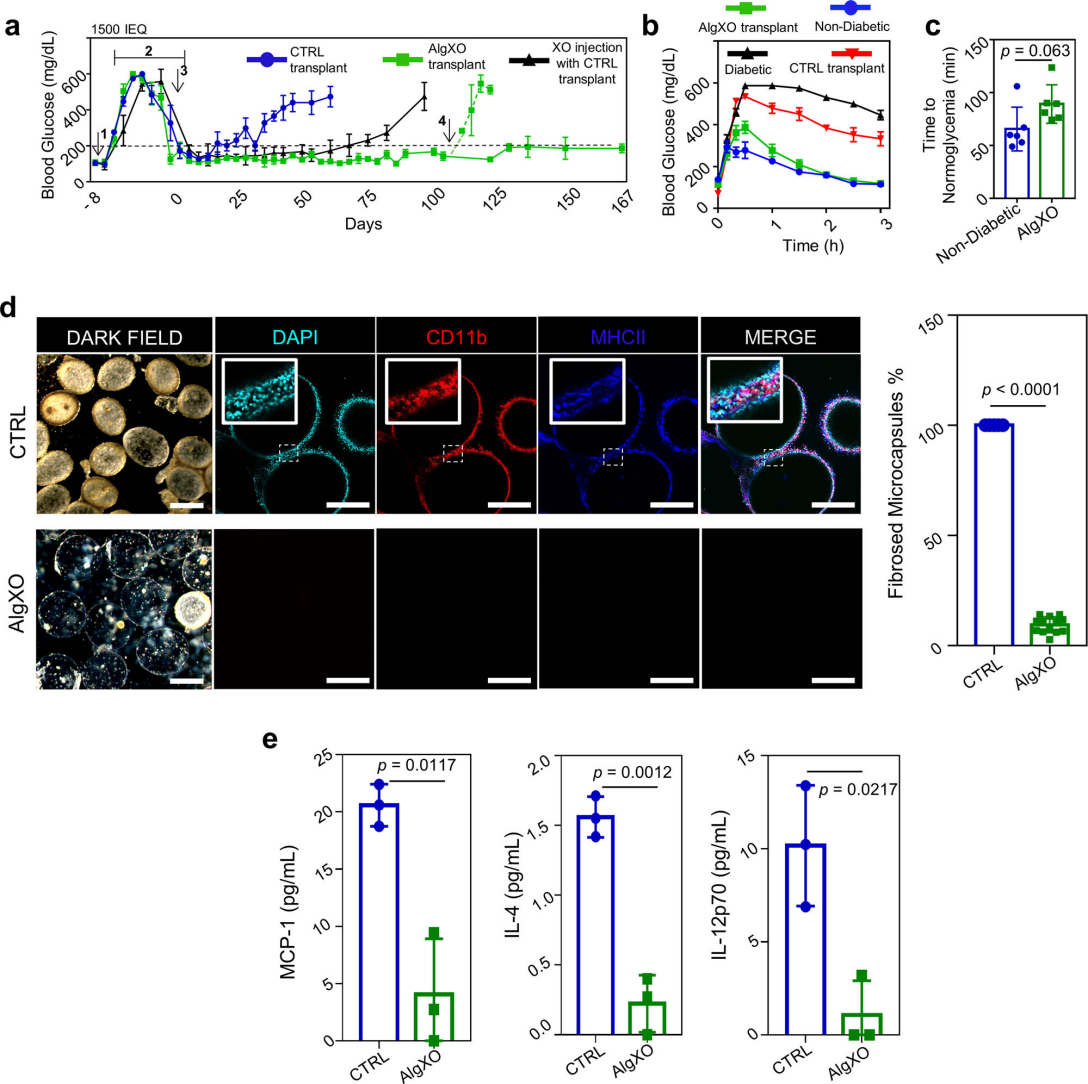
Islet xenotransplants within AlgXO reverse hyperglycemia in diabetic immunocompetent mice. **a** Non-fasting blood glucose levels in C57/BL6 STZ-induced diabetic mice (*n* = 5 mice) shows that transplantation of 1500 IEQ rat islets within AlgXO provided euglycemia in diabetic mice for >170 days, whereas the CTRL microcapsules failed in <1 month. To further confirm that the glycemic correction is merely due to transplants and not pancreatic regeneration in STZ-induced diabetic mice, we washed the i.p. cavity of mice and removed the explants after 105 days of transplantation (*n* = 2 mice). Within 18 h of graft removal, mice blood glucose elevated and remained hyperglycemic for the rest of their lifetime (dashed green line). Separate i.p. transplantation of islets within CTRL microcapsules and XOs provided normoglycemia for ~70 days (black line, *n* = 4 mice). **b** We further tested the efficacy of AlgXO transplants in response to oral glucose tolerance test (OGTT). One month after transplantation, similar to STZ mice (*n* = 6 mice), CTRL microcapsules failed to regulate the glucose levels (*n* = 4 mice), whereas AlgXO transplants successfully reversed hyperglycemia event induced by glucose challenge (*n* = 6 mice), with similar trend as non-diabetic controls. **c** The average time to reach normoglycemia after an OGTT for non-diabetic mice was 65 ± 27 min and for mice with AlgXO transplants was 103 ± 32 min (*n* = 6 mice). **d** After 1 month, both CTRL and AlgXO (from 1500 IEQ group) transplants were removed through washing the i.p. cavity. Next, microcapsules were analyzed for the immune infiltration (also known as pericapsular cell growth) with laser-scanning confocal microscopy. Some cells were CD11b+ and some of the CD11b+ cells were expressing MHCII biomarker. All the collected CTRL microcapsules were found to have pericapsular cells attached to the surface, while the percentage of AlgXO transplants with pericapsular growth was 9.4% ± 3.6%, which was significantly lower than CTRL transplants (*p* < 0.0001). Scale bars are 200 μm for the dark field and 100 μm for the florescent channels. **e** The pericapsular cytokine and chemokines present released in the pericapsular area of implants. Results are mean ± SD, and statistical significance is calculated through unpaired *t*-test with Welch’s correction. 1: STZ injection; 2: Diabetes induction period; 3: Transplantation; 4: Graft removal.

We then sought to investigate the functionality of islet transplantation within AlgXO microcapsules. Rat islets (1500 IEQ, islet equivalent) were encapsulated in either AlgXO or CTRL microcapsules and transplanted into the i.p. cavity of streptozotocin (STZ)-treated C57/BL6 mice with a week-long established hyperglycemia (*n* = 5). To assure the purity and quality of rat islets from each isolation and minimize the batch-to-batch variations between islets, we conducted quality control for every batch (Supplementary Fig. 4). Figure 1a shows that transplantation of rat islets within AlgXO provided euglycemia in diabetic mice for >170 days, whereas the islets transplanted within CTRL microcapsules functionally failed to regulate mice hyperglycemia within a month. To assure that the glycemic correction is due to AlgXO transplants and not beta cell regeneration in diabetic mice, we removed the AlgXO transplants after 105 days of transplantation by washing the i.p. cavity. This date was chosen because not only mice were normoglycemic, but the XO injected groups were hyperglycemic a month prior to it, allowing us to assure the graft function as well as superiority of AlgXO vs XO injected group. Within 16 h of graft removal, the non-fasting blood glucose was elevated and mice remained hyperglycemic (dashed green line, Fig. 1a). To control the effect of AlgXO on the maintenance of hyperglycemia in the STZ-induced diabetic mice, empty AlgXO microcapsules (i.e., without pancreatic islets) were also transplanted into the i.p. cavity of STZ-induced diabetic C57/BL6 mice, but they failed to reverse hyperglycemia (Supplementary Fig. 5). We designed this experiment because a recent study has reported that intravenous injection of UC-MSCs derived XOs into STZ-induced diabetic mice promoted expression and membrane translocation of glucose transporter 4, and reduced the hyperglycemic severity^53^.

We next asked whether the in vivo function of islet xenotransplants could be prolonged by administration of non-encapsulated XOs. We thus transplanted 1500 IEQ rat islets in CTRL microcapsules, and at the same time, injected (i.p.) 8.1 × 10^9^ ± 7.3 × 10^8^ XOs (*n* = 4). This dose was chosen to be consistent with the dose of XOs in AlgXO xenotransplant studies conducted earlier. Administration of non-encapsulated XOs at the time of islet transplantation in CTRL microcapsules extended the euglycemia in diabetic mice for 2 months (Fig. 1a), although not as prolonged as AlgXO transplants. We further tested the efficacy of AlgXO transplants in response to oral glucose tolerance test (OGTT) after 1 month of transplantation (Fig. 1b). After 30 min of glucose challenge onset, the non-diabetic mice blood glucose reached to 291 ± 120 mg/dL (*n* = 4). During the same time, the blood glucose of mice transplanted with islets in AlgXO and CTRL microcapsules were 386 ± 91 mg/dL and 534 ± 9 mg/dL, respectively (*n* = 4). This number was 580 ± 28 mg/dL for diabetic mice (*n* = 3). We set the 200 mg/dL as the normoglycemia threshold between diabetic (hyperglycemic) and non-diabetic (normoglycemic) mice. We then attempted to find the duration required for each mouse to reach normoglycemia after the glucose challenge. Polynomials with degree 5 were assigned to the OGTT curves (Supplementary Fig. 6a, b), and time to normoglycemia was calculated based on the value of 200 for the polynomial functions. Figure 1c demonstrates that 65 ± 27 minutes is the average required time for non-diabetic mice to reach normoglycemia after an OGTT, and such duration was 103 ± 32 min for mice with AlgXO transplants. AlgXO transplants had a slightly higher time to reach normoglycemia after an OGTT compared to non-diabetic controls on average (*n* = 6, *p* = 0.063). This delay is likely due to the barrier of alginate network to the diffusion of insulin and glucose. These results suggest that AlgXO transplants had a comparable glucose response to non-diabetic mice during an OGTT, while none of the CTRL transplants achieved normoglycemia.

Clinical trials for islet transplantation have shown that allogeneic or xenogeneic source of islets affect the clinical efficacy and have led to conflicting results. Although xenotransplantation in non-immunosuppressed diabetic patients partially reduced hypoglycemic events, higher doses of xenogeneic islets were less effective^54,55^. We further performed AlgXO-encapsulated islet dose study to establish a therapeutic dose (Supplementary Fig. 7, Islet Dose Study), where we found that 1500 IEQ rat islets showed longer euglycemic induction than 5000 IEQ, but a lower 500 IEQ failed to correct mice hyperglycemia.

### AlgXO reduces inflammation and fibrosis

We next sought to delineate possible mechanisms that prolonged the function of islet transplants within AlgXO microcapsules. In a broad context, two major players are widely recognized in the long-term failure of microencapsulation technologies: (1) lack of nutrients and oxygen accessibility into the microcapsules, which leads to islet necrosis^15^, and (2) and inflammatory-based foreign body response (FBR) within weeks of transplantation forms a dense fibrotic tissue around the microcapsules, blocking the function of islets^16,17,56^. To investigate possible mechanisms by which islets encapsulated within AlgXO provide longer glycemic correction, we examined both of these points^17–20^.

In the early stage of transplantation, the health and viability of islets suffer from oxidative stress (likely within a week), and at later stages inflammatory-induced fibrosis influences graft viability by constraining oxygen and metabolite diffusion into the microcapsules^17^. Recently, it has been demonstrated that MSC exosomes could relieve the β-cell apoptosis and destruction^53^, and enhance islets survival under hypoxic conditions^57^. Thus, we speculated that XOs may enhance rat islets viability in vitro, and found that XOs (both 20 and 200 μg/mL doses) as well as AlgXO enhance the rat islets viability (Supplementary Fig. 8a, b). It is therefore likely that AlgXO retains the encapsulated islets viability during early stages of transplantation. For longer time periods after transplantation, we sought to understand the viability of islets. Microcapsules from both groups were explanted 1 month after implantation and TUNEL assay was conducted to compare the viability of islets. Supplementary Fig. 8c shows the results of TUNEL assay, where after 1 month of transplantation, the TUNEL positive area of islets transplanted within AlgXO (1.02% ± 0.32%) was higher (*n* = 5, *p* = 0.0256) compared to CTRL (6.44% ± 1.59%). This suggests that after 1 month of transplantation, islets within AlgXO possess higher viabilities.

In later stages of transplantation, however, inflammatory-led FBR further compromise the viability and functionality of the islets within microcapsules. Inhibition of inflammation-led fibrosis has been shown to improve long-term function of transplanted islets and euglycemia in diabetic rodents^1,17,18,24^. Recognizing the multi-potent anti-inflammatory properties of MSCs derived XOs^43,58,59^, we hypothesized that encapsulation of rat islets within AlgXO microcapsules reduces the inflammatory response, leading to the long-term function of islets and glycemic control in immunocompetent diabetic mice. To investigate the inflammatory response against AlgXO and CTRL xenotransplants, we explanted both groups and analyzed for immune infiltration.

Since the 1500 IEQ CTRL transplants failed to function within about 1 month (Fig. 1a), we explanted CTRL and AlgXO xenotransplants from mice at day 31 of the implantation. We next analyzed the pericapsular attachment around both microcapsules and observed that CTRL groups are covered with CD11b+ cells, while most of AlgXO explants were clear and transparent (Fig. 1d). It is noteworthy that 9% ± 3.6% of the microcapsules from AlgXO explants showed pericapsular cell attachment that was significantly lower than pericapsular cell attachment on CTRL explants (Fig. 1d, *p* < 0.0001). Analyses of the subtypes that infiltrated around CTRL microcapsules revealed the presence of CD11b+ myeloid-derived cells. At least in some locations, CD11b+ cells express MHCII+, as observed through co-localization of CD11b and MHCII markers. As one of the main myeloid-derived cells, macrophages generally express moderate levels of MHCII to regulate immune tolerance and local surveillance to maintain homeostatic immunity. However, macrophages will upregulate MHCII expression and antigen presentation capacity in a pro-inflammatory environment, where antigens can be presented to CD4+ lymphocytes.

To better understand the effect of XOs on the pericapsular environment, we explanted the grafts 1 month after transplantation and analyzed the lavage solution obtained from the explants. Analyses of cytokines and chemokines demonstrated reduced secretion of MCP-1 (20.6 ± 1.8 pg/ml to 4.1 ± 4.96 pg/ml, *n* = 3, *p* = 0.0117), IL-4 (1.6 ± 0.2 pg/ml to 0.2 ± 0.2 pg/ml, *n* = 3, *p* = 0.0012), and IL-12p70 (6.7 ± 6.6 pg/ml to 1.1 ± 1.8 pg/ml, *n* = 3, *p* = 0.0217) in the pericapsular area of AlgXO transplants compared to controls (Fig. 1e). MCP-1 mediates the recruitment of inflammatory monocytes to the site of inflammation and IL-12p70 These results in their totality suggest less recruitment of immune cells to the AlgXO compared to CTRL microcapsules.

Next, we sought to further understand the mechanisms for the observed differential inflammatory responses against AlgXO and CTRL transplants. Alginate immunogenicity has been attributed to two separate mechanisms, which could also be viewed as complementary phenomena. First, prior studies have shown endotoxin contaminations within alginate are the main immunogens, including lipopolysaccharide (LPS), lipoteichoic acid, and peptidoglycans^60,61^. Through lack of endotoxin presence within the commercially purified alginate (such as the UPLVG in the present study), others have reported that even without endotoxins alginate may enhance immune response^16,24^. We recently have shown that even an ultrapure alginate could stimulate macrophages to an inflammatory lineage^62^. These reports suggest that such inflammatory response is likely due to the inherent nature of alginate. Guluronate oligosaccharide derived from alginate, for example, has been reported to readily activate macrophages partly through Toll-like receptor 4 (TLR4) signaling pathway^62,63^. While the exact mechanisms for such response is debated, resolving the inflammatory response against alginate microcapsules is unanimously reported to prevent or delay the fibrosis. In this context, many groups have reported the long-term efficacy of islet transplantation within fibrosis-resistant devices^18,24,26,27^.

**Fig. 2:**
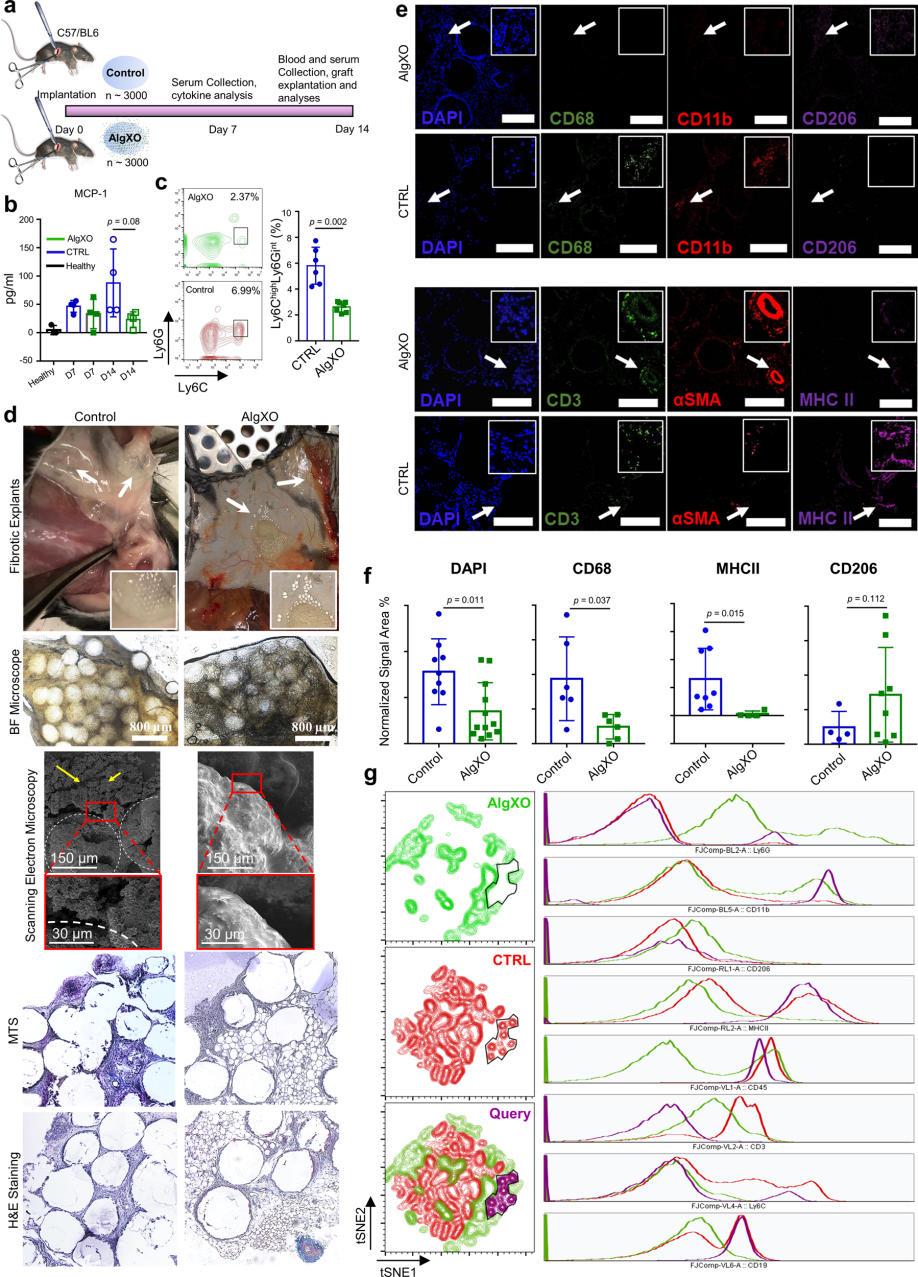
AlgXO microcapsules show reduced inflammatory response after 2 weeks transplantation. **a** Study design to compare and quantify the inflammatory response against AlgXO and CTRL microcapsules. A 2-week study was performed since this timeframe is suitable for resolving and reflecting both innate and adaptive immune system as well as fibrotic responses to implanted materials in C57/BL6 mice. Blood was collected on days 7 and 14 for immunocytes and inflammatory cytokines analyses (*n* = 4 mice). **b** Two weeks after implantation, MCP-1 chemokine was 3.7-fold less in the bloodstream of mice that had received AlgXO versus the ones transplanted with CTRL. **c** CD45 + CD11b + Ly6C^high^Ly6G^med^ inflammatory monocytes were significantly lower (*p* = 0.002) in the bloodstream of mice transplanted with AlgXO compared to CTRL (*n* = 3 mice). **d** While captured images from explants and their BF microscopy are similar, sections and scanning electron micrographs from two weeks explants show different immune environment around microcapsules. White arrows show the localization of grafts and yellow arrows point to the cells infiltrated around microcapsules. **e**, **f** Fixed fibrotic tissues were later sectioned and stained for subpopulations of immunocytes. Normalized areas of DAPI, CD68, and MHCII around explant microenvironments of AlgXO were significantly lower than CTRL microcapsules (*n* = 4 mice). **g** Total cells of fibrotic tissues were isolated and stained for flow cytometry analyses of immunocytes subpopulation. tSNE plots further demonstrate the different immune environment around AlgXO and CTRL explants. We further conducted a query on a subpopulation that is present on CTRL but absent in AlgXO immune environment. This subpopulation is CD45 + CD11b + CD19 + MHCII + CD3−Ly6C−, which is likely to be the memory B cells sob-population. Results are mean ± SD, and statistical significance is calculated through unpaired *t*-test with Welch’s correction.

To comprehensively compare the inflammatory response of AlgXO and CTRL microcapsules, we further focused on the empty microcapsules and the inflammatory response they induce in vivo. We transplanted ~3000 AlgXO or CTRL microcapsules into the subcutaneous space of C57/BL6 mice, and both microcapsules were explanted after 2 weeks (Fig. 2a). A 2 weeks timepoint was selected, since it has been established as a suitable timepoint to resolve and reflect both innate and adaptive immune system as well as fibrotic responses to implanted materials in C57/BL6 mice^16,64^. At days 7 and 14 post-transplant serum cytokines were also measured, reflecting the systemic inflammatory response, if any. Among 11 cytokines examined, there was no significant difference between serum cytokines of mice that were subcutaneously transplanted with AlgXO or CTRL (Fig. 2b and Supplementary Fig. 9). While not statistically significant, the average amount of MCP-1 chemokine in the serum of mice transplanted with CTRL microcapsules for 2 weeks (87.8 ± 59.9 pg/mL) was 3.7-fold higher than mice implanted with AlgXO microcapsules (23.3 ± 13.4 pg/mL). The difference between systemic MCP-1 led us to further study the circulatory inflammatory monocytes in response to AlgXO and CTRL transplants.

Detection of circulating microbial molecules or pro-inflammatory cytokines by bone marrow-resident cells leads to MCP-1 production to modulate the frequency of circulating inflammatory monocytes^65^. MCP-1 is a chemokine that binds to CCR2 and mediates the recruitment of inflammatory (Ly6C^high^) monocytes to the site of inflammation. We next sought to quantify the inflammatory monocytes in the mice’s blood 2 weeks after implantation. Figure 2c shows the flow cytometry plots and their quantification of the inflammatory monocytes (CD45 + CD11b + Ly6C^high^Ly6G^med^)^66^ subpopulation. CD45 + CD11b + Ly6C^high^Ly6G^med^ in the mice transplanted with AlgXO (2.62% ± 0.4%) were significantly lower (*n* = 3, *p* = 0.002) compared to those monocytes in the blood circulation of CTRL microcapsules (5.8% ± 1.4%). These observations suggest that the AlgXO transplants are likely to reduce the systemic inflammatory response against alginate microcapsules.

The destination of circulating monocyte has been linked to the site of MCP-1 secretion^67,68^, which is also a site of hyper-inflammation. In the present study, the transplant site is likely to be the main site of inflammatory response^62^. We therefore sought to investigate the local inflammatory response around the implants 2 weeks after transplantation. We found that all detectable CTRL microcapsules had agglomerated into clumps of alginate aggregates (Fig. 2d). For AlgXO, while some microcapsules were remained intact and non-aggregated (shown with white arrows), the rest were entrapped in a pseudo tissue with multiple blood vessels around them. These tissues from both groups were isolated carefully so as not to contain endogenous tissues of mice. Bright Field microscopy demonstrated that pseudo tissues have entrapped microcapsules (Fig. 2d). Under scanning electron microscopy evaluations, some microcapsules were detected (Fig. 2d; white dashed line for visual guide of microcapsules) were surrounded with rough microstructures and the AlgXO ones were entrapped in smooth structures. Tissues were sectioned into 5–10-μ slices and stained with H&E and Masson’s trichrome staining (MTS). The fibrotic tissue formed around CTRL microcapsules demonstrated the significant infiltration of mostly mononuclear cells, while such histology was not observed in AlgXO fibrotic tissues (Fig. 2d).

To better compare the immune environment, we compared and quantified the cellular components that drive the fibrotic response. Both fibrotic tissues were stained for different immunocytes including macrophages (CD11b+ and CD68+), T cells (CD3+), pro-regenerative macrophages (CD206), antigen-presenting cells (MHCII), and fibrotic marker of smooth muscle actin (αSMA). DAPI counterstaining was also used to count total cell infiltration within fibrotic tissues (Fig. 2e, and Supplementary Fig. 10). Figure 2f shows that total cell infiltration around microcapsules was significantly lower in AlgXO fibrotic tissues (*p* = 0.011). Similar trends were observed for CD68 (*p* = 0.037) and MHCII (*p* = 0.015). In contrast, there was no association between CD206 expression (*p* = 0.112). We further discussed these results in Supplementary Information under Immune Microenvironment Around Subcutaneous Microcapsules section.

To gain more holistic information on the fibrotic tissues around CTRL and AlgXO microcapsules, we compared the components of both microcapsules at the cellular level using flow cytometry. The tSNE plots in Fig. 2g demonstrate highly segregated subpopulations for AlgXO and CTRL fibrotic tissues. We particularly queried the subpopulations that were absent in AlgXO but present in CTRL as shown in Fig. 2g black line area (purple colored query). This subpopulation is CD45 + CD11b + CD19 + MHCII + CD3−Ly6C−, which is likely to be the memory B cells subpopulation. To further assess the quantity of B cells in fibrotic microenvironments, we analyzed CD45 + CD19+ B cells (Supplementary Fig. 11a). The CD45 + CD19+ cells were remarkably fewer (*n* = 4, *p* < 0.0001) in AlgXO (0.9% ± 0.5%) compared to that of CTRL (22.5% ± 5.1%). B lymphocytes play critical roles in the FBR against alginate microcapsules. In particular, genetic deletion of B cells as well as CXCL13 neutralization have been reported to dampen the FBR to implanted alginate microcapsules during a 2-weeks implantation period^16^, which aligns with our observations in the present study. In addition to B cells, innate lymphoid cells and γδ^+^ T cells lead to a chronic adaptive antigen-dependent Th17 cell response^69^. In our study, we found that there was a higher quantity of CD3+ in AlgXO compared to CTRL (*p* = 0.026), which is likely due to the blood/blood vessels in the AlgXO microenvironment (Supplementary Fig. 11b). This could be further confirmed due to the vicinity of blood vessels with T cells (Fig. 2e). While inflammatory response was reduced around subcutaneous AlgXO microcapsules, transplantation of 155 IEQ rat islets within the subcutaneous space of diabetic mice did not restore the euglycemia (Supplementary Fig. 13), which is likely due to the observed fibrotic response.

### AlgXO’s reduced foreign body response is partly due to the releasing of exosomes in a controlled fashion

**Fig. 3:**
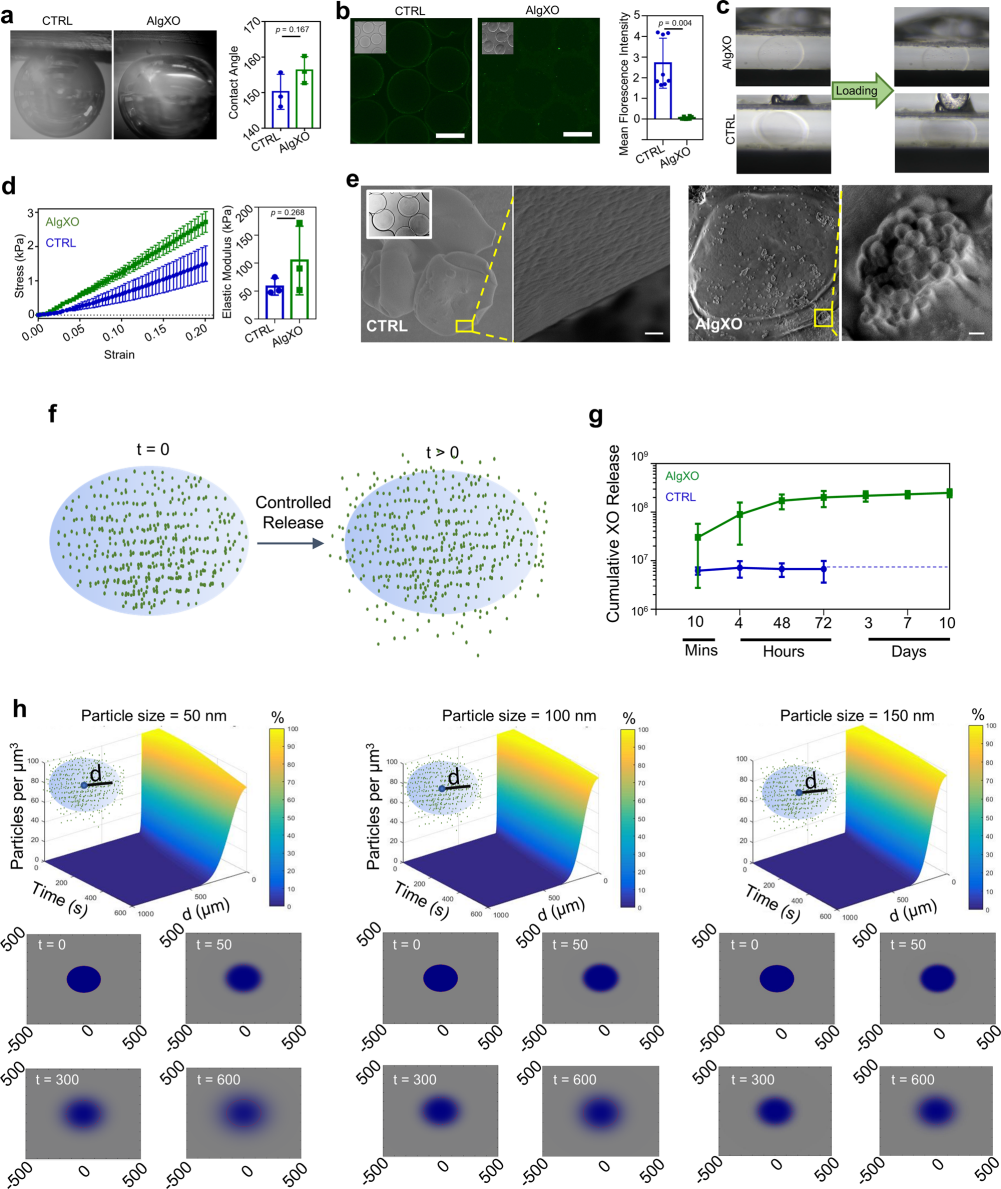
AlgXO reduces FBR partly due to releasing XOs in a controlled fashion. **a** A non-significant difference between AlgXO’s and CTRL’s captive bubble contact angle was observed (i.e., 156.3° ± 3.8° for AlgXO versus 150.2° ± 4.9° for CTRL). **b** IgG protein adsorption to the surface of AlgXO and CTRL microcapsules (*n* = 3, i.e., three different microcapsule fabrication). Microscale mechanical testing was performed for **c** AlgXO and CTRL. **d** Stress–strain curve of AlgXO vs. CTRL microcapsules graphed based on the force-displacement data. Elastic modulus was not significantly different (*p* = 0.268) between AlgXO (104.7 ± 61.4 kPa) and CTRL (57.8 ± 14.9 kPa). **e** to investigate the release of encapsulated exosomes, scanning electron microscopy was conducted on air-dried microcapsules, demonstrating surface pores in 50–200 nm size scale (scale = 1 μm), and encapsulation of vesicles within AlgXO. **f** schematic representation of our hypothesized model, where XOs release from AlgXO microcapsules overtime. **g** AlgXO releases XOs in a control fashioned in vitro. Release profile reaches a threshold within a week. **h** diffusion of nanoparticles with diameters 50, 100, and 150 nm (which are chosen due to the size ranges of XOs). Particles number vs. time vs. distance from microcapsule center (**d**) are graphed in the percentage heatmap diagrams. Bottom panels show the color map of spatiotemporal diffusion rate of XOs at *t* = 0, 50, 300, and 600 s upon initiation of diffusion. Expectedly, the smaller the size, the higher the diffusion rate. In addition, 600 s after the onset of diffusion, the concentration of 50 nm particles at the center of the microcapsules dropped 20%, while for 100 and 150 nm, no significant decrease was obtained at the center of the microcapsules. Results are mean ± SD, and statistical significance is calculated through unpaired *t*-test with Welch’s correction.

We pursued our investigation to delineate the effect of XOs controlled release on the AlgXO’s reduced inflammatory response. We first characterized the physical and mechanical properties of AlgXO and compared them against CTRL microcapsules, as these properties remarkably influence the biological response of biomaterials. Water interactions with biomaterial surface, for example, have been recognized as a fundamental characteristic determining the immunological responses of biomaterials. Compared to hydrophilic materials, hydrophobic (and slightly hydrophilic) biomaterials adsorb more proteins. This is mainly because proteins adjacent to hydrophilic surfaces must displace more water molecules bound to biomaterial surface^21,70^. We thus sought to measure the contact angle for AlgXO and CTRL biomaterials using captive bubble contact angle method. Figure 3a shows that the contact angle for AlgXO (156.3° ± 3.8°) was higher than that of CTRL microcapsules (150.2° ± 4.9°). This suggests that AlgXO is slightly more hydrophobic; however, there was no significant correlation (*n* = 3, *p* = 0.167). Importance of hydrophobicity lies in protein adsorption onto the biomaterials surface, which has been linked with the FBR. Many studies have reported that the blocking of protein adsorption of biomaterials silences the immune response^24,71^. These proteins may include components of the coagulation cascade (fibrinogen and tissue factors), complement cascade (C5), and other plasma-derived proteins (albumin and IgG)^72^. IgG and fibronectin adsorption led the Mac-1-mediated attachment of neutrophils and macrophages to biomaterial surfaces during the acute phase of inflammation^73^. We therefore attempted to investigate the IgG adsorption onto both AlgXO and CTRL microcapsules. IgG adhered to the surface of CTRL microcapsules more pronouncedly (2.70% ± 1.21%, *n* = 3, *p* = 0.0004) compared to AlgXO (0.05% ± 0.06%) (Fig. 3b). The less-fibrotic properties AlgXO could partly originate from the less protein adsorption onto its surface. Proteins absorption onto the biomaterials and their conformation could lead to the formation of different biomaterial-associated molecular patterns, initiating the inflammatory response^74^.

Mechanical properties of biomaterials have been linked to immunological response of implants. For instance, macrophage confinement reduces their inflammatory response through reduction in actin polymerization and LPS-stimulated nuclear translocation of MRTF-A^75^. In addition, macrophages adhere to stiff surfaces more profoundly^76^. We thus attempted to characterize the mechanical properties of both AlgXO and CTRL microcapsules (Fig. 3c, d). Figure 3c demonstrates the images of the initial and final vertical positions of the cantilevers, exerting pressure on the microcapsules. Stress–strain curves (Fig. 3d) shows the linear behavior, where the difference between elastic modulus of AlgXO (104.7 ± 61.4 kPa) and CTRL (57.8 ± 14.9 kPa) was not significant (*n* = 3, *p* = 0.268).

We further asked other possible mechanisms that are likely to play roles in AlgXO’s immunomodulatory properties. Our initial hypothesis was that the immunomodulatory effects of AlgXO is partly due to the release of XOs. MSC-derived XOs have been demonstrated to possess immunosuppressive functions both in vitro^77^ and in rodent models^46^. To better understand this possibility, we sought to find whether XOs within AlgXO release into the surrounding microenvironment of microcapsules. We first visualized and compared the CTRL and AlgXO microcapsules using Scanning electron microscopy (SEM) on air-dried microcapsules. Figure 3e demonstrates SEM micrographs of both AlgXO and CTRL microcapsules, suggesting the presence of surface pores in the 50–200 nm size scale. SEM micrographs of AlgXO microcapsules demonstrated the encapsulation of spherical-shaped vesicles within AlgXO, and their possible release from the surface of a microcapsule (Fig. 3e, right panel, scale = 1 μm). We particularly hypothesized that XOs could be released from AlgXO (Fig. 3f) as they readily diffuse within the nano-meshes of extracellular matrix and communicate over long distances within the body. Recently, it has been demonstrated that due to the aquaporin-1 mediated XOs deformability, XOs could transport within and diffuse outwards of alginate matrix (as well as extracellular matrices), despite XOs being larger than the mesh size of the surrounding network^78^. We next incubated AlgXO microcapsules in vitro and measured the release of XOs, demonstrating the controlled release of XOs over the course of 10 days (Fig. 3g). We found that 3.03 × 10^07^ ± 2.75 × 10^07^ XOs are released within 10 min of culturing, and total release increases to 2.49 × 10^08^ ± 4.63 × 10^07^ XOs within 10 days. It should be noted that total XOs encapsulated is 5.43 × 10^09^ ± 4.84 × 10^09^ (Supplementary Fig. 2b). To further understand the release profile of XOs, we modeled the release of exosomes based on the Fickian diffusion of nanoparticles ranging from 50 to 150 nm in diameter (i.e., the size range of XOs) (Supplementary Information). Figure 3h demonstrates the simulated diffusion of XOs with 50, 100, and 150 nm. The top panels are time (s) *vs*. particles concentration (per μm^3^) vs. distance from capsules center (μm). Bottom panels on Fig. 3h demonstrate the heatmap representation of the XOs diffusion outwards of microcapsules. In these maps the 1 mm × 1 mm diffusion microenvironment is shown, and the blue color represents the diffusion. These simulations suggest that smaller particles (50 nm of diameter) diffuse faster than 150 nm particles. To check our simulation models beyond the scale of 50–150 nm, smaller (i.e., 10 nm) and larger (200 and 500 nm) particles were input into the code. It was found that in 600 s, while 10 nm possess expedited diffusion rates, 500 nm particles remain within the microcapsules and no outward diffusion was obtained (Supplementary Fig. 14).

### XOs suppress murine macrophages and T lymphocytes

**Fig. 4:**
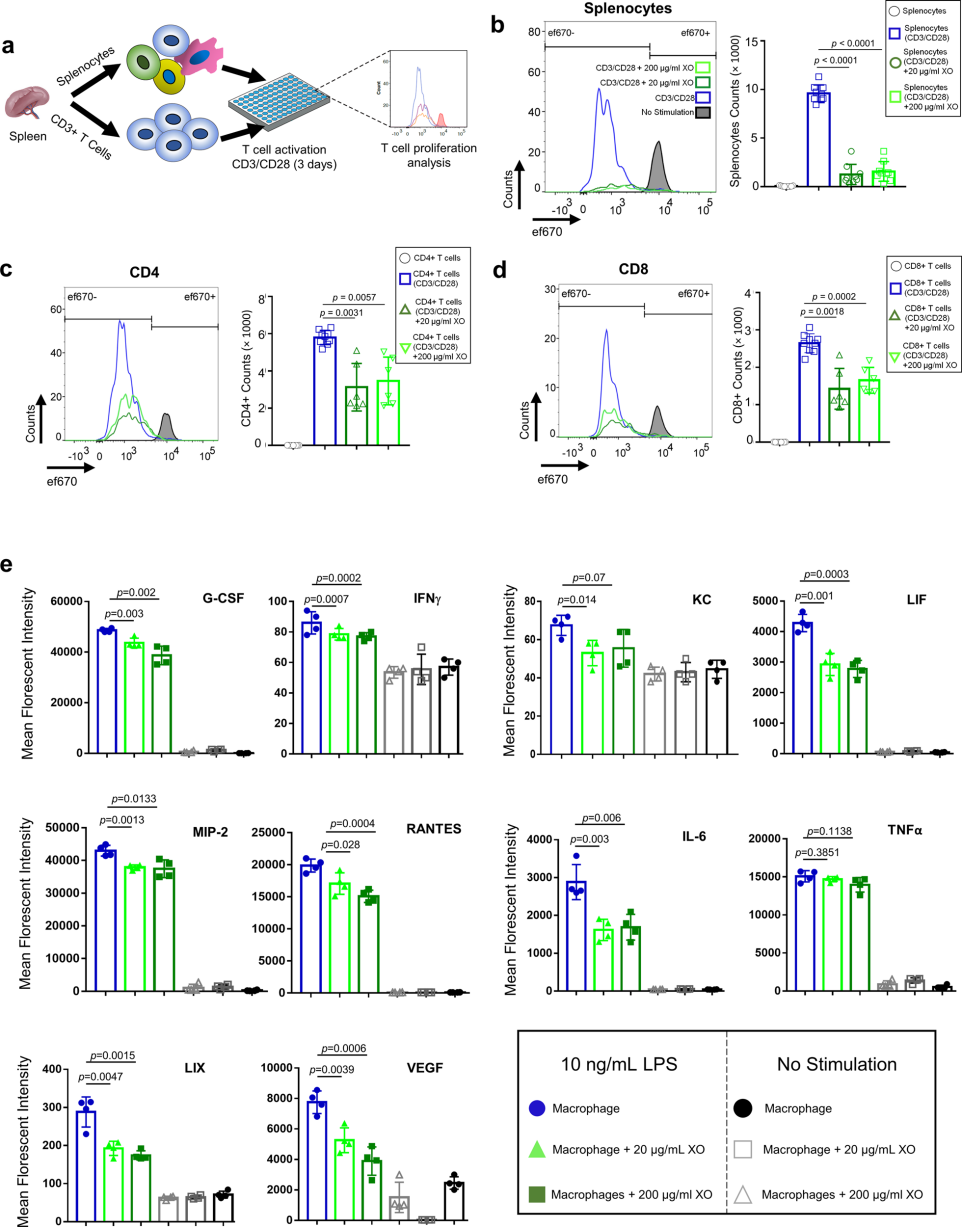
XOs suppress the proliferation of splenocytes and CD3+ T cells and reduce the production of inflammatory cytokines from LPS-stimulated macrophages. **a** Schematic figure showing the experimental procedure. CFSE-labeled splenocytes and CD3+ T cells were co-cultured with plate-bound anti-CD3 and soluble CD28 in the presence or absence of 20 and 200 μg/mL of XOs. Upon 4 days co-culture, cells were analyzed using flow cytometry. **b** Splenocyte counts for CD3/CD28 activated cells were 9603 ± 871, and addition of 20 and 200 μg/mL XOs reduced the counts to 1253 ± 1038 (*n* = 4, *p* < 0.0001, 4 mice) and 1570 ± 1010 (*n* = 4, *p* < 0.0001), respectively. **c** In the co-cultures of CD3+ cells with CD3/CD28 antibodies, CD4+ counts for CD3/CD28 activated T cells was 5217 ± 378. Addition of 20 and 200 μg/mL XOs reduced the counts to 3889 ± 2081 (*n* = 4, *p* = 0.0031, 4 mice) and 4387 ± 1397 (*n* = 4, *p* = 0.0057, 4 mice), respectively. **d** In the co-cultures of CD3+ cells with CD3/CD28 antibodies, CD8+ counts for CD3/CD28 activated T cells was 2700 ± 252. Addition of 20 and 200 μg/mL XOs reduced the counts to 1503 ± 784 (*n* = 4, *p* = 0.0018, 4 mice) and 1766 ± 628 (*n* = 4, *p* = 0.0002), respectively. **e** Addition of XOs to the co-cultures of murine macrophages reduces the secretion of inflammatory cytokines (G-CSF, IFNγ, IL-6, LIF, LIX, MIP-2, RANTES) in a dose-dependent manner (*n* = 4, biological replicates). Results are mean ± SD, and statistical significance is calculated through unpaired *t*-test with Welch’s correction.

Similar to the suppressive properties of their parental cells, XOs derived from MSCs have been demonstrated to possess immunosuppressive functions both in vitro^77^ and in rodent models^46^. We recently showed that bone marrow-derived MSC XOs suppress human peripheral blood mononuclear cells (PBMCs) upon activation with anti-CD3/CD28 stimulation^43^. In the splenocyte co-cultures supplemented with IL-2, bone marrow-derived MSC XOs also induced CD4 + CD25 + FoxP3+ regulatory T cells^43^. To understand the mechanisms by which XOs (derived from umbilical cords) exert their suppressive function, here we first studied their effects on activated murine splenocyte and then on purified CD3+ T cells isolated from splenocytes (Fig. 4a). Cell Proliferation Dye eFluor670-labeled splenocytes from C57/BL6 wild-type mice were stimulated with plate-bound anti-CD3 and anti-CD28 in vitro in the presence and absence of XOs. Both 20 and 200 μg/mL XOs suppressed the splenocytes proliferation, where activated splenocytes proliferated to the number of 9603 ± 871, and addition of 20 and 200 μg/mL XOs reduced the counts to 1253 ± 1038 (*n* = 4, *p* < 0.0001) and 1570 ± 1010 (*n* = 4, *p* < 0.0001).

The cellular heterogeneity within splenocytes complicates the drawing of conclusion on the XOs cellular mechanism. To delineate more detailed cellular mechanisms underlying suppressive capabilities of XOs, we focused on the XOs effect on the activation of purified T cells. We thus repeated the T cells proliferation assay in purified T cells co-cultures, which gives insight onto the interactions between XOs and T lymphocytes. Purified CD3+ T cells were activated (similar to splenocyte activation procedure), and after 4 days the CD4+ counts for CD3/CD28 activated T cells was 5217 ± 378. Addition of 20 and 200 μg/mL XOs reduced the counts to 3889 ± 2081 (*n* = 4, *p* = 0.0031) and 4387 ± 1397 (*n* = 4, *p* = 0.0057), respectively. Moreover, CD8+ counts for CD3/CD28 activated T cells was 2700 ± 252 and addition of 20 and 200 μg/mL XOs reduced the counts to 1503 ± 784 (*n* = 4, *p* = 0.0018) and 1766 ± 628 (*n* = 4, *p* = 0.0002), respectively. Interestingly, XOs were more suppressive in the splenocytes co-cultures than purified T cells. These results suggest the remarkable involvement of non-T cells, including antigen-presenting cells (APCs), in the XOs suppressive mechanism in splenocyte co-cultures. This suggests that XOs, at least in part, target accessory cells such as APCs rather than T cells directly, which is in agreement with recent studies^58,79^. In a broader context, infused MSCs and their apoptotic products are suggested to be phagocytosed, leading to the generation of third-party phagocytes that ultimately mediate the observed immunomodulatory effects^80^. These observations imply that XOs first interface with APCs and phagocytes^46,58^, which then facilitate the immunosuppression of T cells. These observations are in agreement with our earlier in vivo results, where CD3+ T lymphocytes were absent in the lavage collected from the AlgXO microcapsules microenvironment, but were present in the lavage collected from the microenvironment of CTRL microcapsules (Supplementary Fig. 12d).

To functionally validate whether XOs possess immunomodulatory effects on APCs, and gain insight into XOs therapeutic mechanisms, we performed co-cultures of activated murine macrophages and XOs. Recently, the anti-inflammatory potentials of XOs derived from human-derived MSCs have been described in the LPS induced inflammation both in vitro cultures with murine macrophages and LPS injected mouse models^77^. To gain insight into the possible mechanisms that XOs regulate macrophages activation, we isolated the supernatants from co-cultures and measured the quantity of secreted cytokines. Among the panel of tested cytokines, we found that XOs significantly reduce the production of G-CSF, IFNγ, LIF, KC, MIP-2, RANTES, IL-6, LIX, and VEGF from LPS-stimulated macrophages (Fig. 4e). LPS activates the NFκB pathway and all three MAPK pathways (ERK, JNK/SAPK, and p38α), leading to a wide range of cellular responses, including cell differentiation, survival or apoptosis, and inflammatory responses^81^. Reduced cytokines and chemokines in macrophage culture are hallmarks of NFκB inflammatory pathway, suggesting that XOs likely possess anti-inflammatory properties through regulating this pathway (see Supplementary Table 1). Inflammatory cytokines/chemokines that were not affected by XO addition include TNFα, IL-2, IL-17, and IL-1a (Supplementary Fig. 15). Interestingly, even the production of IL-10 was reduced by addition of XOs, demonstrating that in this specific experimental setting and timepoints, XOs have immunosuppressive roles.

### XOs suppress human T lymphocytes and regulate NFκB in human macrophages

**Fig. 5:**
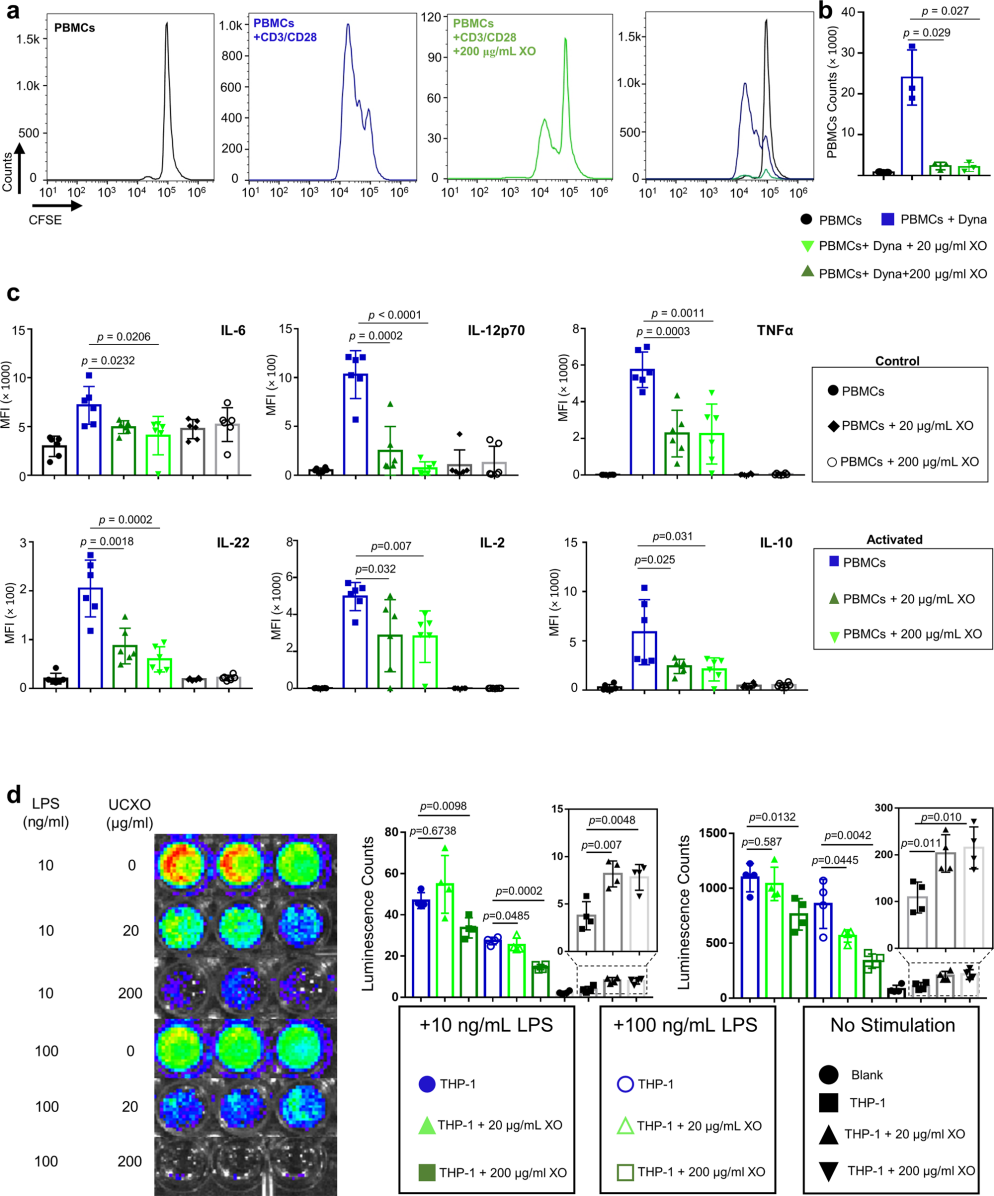
XOs suppress human peripheral blood mononuclear cells and macrophages. **a** Human peripheral blood mononuclear cells (PBMCs) were activated with bead-bound CD3/CD28 antibodies in the presence and absence of XOs. **b** Addition of 20 and 200 μg/mL XOs reduced the count of activated PBMCs from 24,002 ± 6762 to 2342 ± 910 (*n* = 3; *p* = 0.029, 3 different donors) and to 2102 ± 1121 (*n* = 3; *p* = 0.027, 3 different donors), respectively. To gain more insight into the XOs mechanism of action, cytokine production was evaluated in the PBMCs culture. Addition of XOs reduced the IL-6, TNFα, IL-12p70, and IL-22 production from activated PBMCs. **c** In the co-cultures of anti-CD3/CD28 activated PBMCs, addition of XOs reduce IL-2, IL-6, IL-10, IL-12p70, IL-22, and TNFα (*n* = 3, 3 different donors). **d** XOs suppressed the LPS mediated human macrophages activation. LPS activated NFκB pathway in THP-1 macrophages, and addition of 200 μg/mL of XOs reduces NFκB activation of both 10 ng/mL LPS (*n* = 4, *p* = 0.044, 4 biological replicates) and 100 ng/mL LPS (*n* = 4, *p* = 0.004, 4 biological replicates) activated THP-1 macrophages. 20 μg/mL of XOs was not enough to interfere with the NFκB activation. XOs influenced the NFκB activation of non-activated THP-1 cells. Addition of 20 μg/mL XOs upregulated the NFκB activity in THP-1 cells from 109 ± 17 to 203 ± 20 (*n* = 4, *p* = 0.0117, 4 biological replicates). Furthermore, addition of 200 μg/mL XOs upregulated the NFκB activity in THP-1 cells from 109 ± 17 to 215 ± 23 (*n* = 4, *p* = 0.0105, 4 biological replicates). Results are mean ± SD, and statistical significance is calculated through unpaired *t*-test with Welch’s correction.

Our in vivo and in vitro assays thus far demonstrated the xenogeneic immunosuppressive capabilities of XOs. We further asked the replicability of such immunosuppressive potency of XOs on human-derived immunocytes, i.e., an allogeneic response. Since we observed a reduction of inflammatory response and an induction of tolerance in AlgXO transplanted mice in vivo, and reduction of murine T-cell proliferation and macrophage activation, we attempted to understand the immunomodulatory effects of XOs on human-derived immune cells in vitro. We examined XOs suppressive activity on T-cell proliferation ex vivo using carboxyfluorescein succinimidyl ester (CFSE)-labeled human peripheral blood mononuclear cells (PBMCs). PBMCs were activated with bead-bound anti-CD3/CD28 (1:1 ratio) and further cultured with or without XOs. Both 20 and 200 μg/mL XOs suppressed activation of PBMCs (Fig. 5a). Quantitatively, addition of 20 and 200 μg/mL XOs reduced the count of activated T cells from 24002 ± 6762 to 2342 ± 910 (*n* = 3; *p* = 0.029) and to 2102 ± 1121 (*n* = 3; *p* = 0.027), respectively (Fig. 5b). These results are consistent with previous studies where the ability of MSC-derived exosomes to suppress T-cell activation and proliferation was reported^43,51,59^. These results collectively suggest that XOs have potent suppressive effects on T cells activation, although the mechanisms behind such suppression remain to be fully understood.

To gain a better understanding on the underlying cellular pathways, we performed Luminex assay to measure some cytokine profiles in the supernatant of PBMC co-cultures (Fig. 5c and Supplementary Fig. 16). We particularly examined cytokines that are related to macrophages and pro-inflammatory T lymphocyte subsets, such as Th1 and Th17 lymphocytes that play key roles in the FBR against biomaterials^69,82^. In addition, recent reports have demonstrated the suppressive effects of XOs on Th1/Th17 cells polarization both in vitro and in vivo^43,51,59^. To mechanistically probe the XOs effect in inhibiting the induction of T cell to Th1/Th17 subtypes, we measured several key representative Th1 and Th17 cytokines. In the presence of XOs, the levels of several pro-inflammatory Th1 and Th17 cytokines including IL-12p70 (Th1), TNFα (Th1), IL-6 (Th17), and IL-22 (Th17) were significantly reduced (Fig. 5c). IFNγ (Th1) demonstrated a trend of decrease though not significant (Supplementary Fig. 16). Interestingly, XOs significantly reduce the production of IL-2, which is a key cytokine to stimulate the growth, proliferation, and differentiation of T lymphocytes.

Cytokines are generally recognized as “signal 3”, which polarize helper T cells to Th1 (e.g., by IL-12 exposure) or Th17 (by IL-6 and IL-23) subsets. In addition, cytokines play a fundamental role in clonal expansion and persistence of antigen-reactive T lymphocytes and their effector activity. For instance, IFN-γ, IL-12, and IL-23 bind onto their receptors expressed on naive CD4+ T cells and drive the differentiation of Th1 cells through the activation of signal transducer and activator of transcription 1 (STAT1), STAT4, and T box transcription factor (T-bet)^83,84^. Moreover, TNF–TNFR pairs control T-cell responses in two ways. First, they provide proliferative and survival signals either directly to the T cells or to the cognate APCs, regulating the frequency of effector and/or memory CD4+ or CD8+ T cells that can be differentiated from naive T cells in response to antigen stimulation. Second, they control T-cell function directly by promoting the production of cytokines such as IL-4 and IFNγ, or indirectly through stimulating the production of pro-inflammatory cytokines, such as IL-1 and IL-12, by professional or non-professional APCs^85^. Upregulated IL-6 binds onto its receptor and activates retinoid-related orphan receptor γ T (RORγt) and STAT3, driving Th17 cell differentiation and function^84,86^. Pathogenic Th17 cells are then polarized as a result of IL-23 and TGFβ3 stimulation^87^.

We and others have observed that MSCs induced Treg expansion in a trans-well system only in the presence of splenocytes or peripheral blood monocytes, but not with purified CD4+ T cells^43,88–90^. Exosome-treated monocytic THP-1 (but not MyD88-deficient THP-1) cells polarized activated CD4+ T cells to CD4 + CD25 + FoxP3+ Tregs at a ratio of one exosome-treated THP-1 cell to 1000 CD4+ T cells^91^. It is likely that XOs (as well as MSCs) play their immunosuppressive roles through interacting with myeloid lineage, and indeed, adoptive transfer of macrophages or monocytes, treated with MSC-EVs in vitro can protect the lung from injury^92^. We next sought to gain insight into the mechanisms by which XOs suppress macrophage activation by employing macrophages that produce luciferase in response to NFκB activation. Figure 5d shows the representative images of the luciferase activity (NFκB activity) as a result of 10 and 100 ng/mL LPS stimulation in the presence and absence of XOs. Luminescence count from IVIS imaging was quantified using equivalent regions on interest, suggesting that addition of 200 μg/mL of XOs reduces NFκB activation of both 10 ng/mL LPS (*p* = 0.044) and 100 ng/mL LPS (*p* = 0.004) activated THP-1 macrophages. Interestingly, 20 μg/mL of XOs did not efficiently reduce the NFκB activation. Same conditions were replicated, and signals were acquired using a plate reader, demonstrating a similar trend in the potency of XOs to inhibit NFκB activation (Fig. 5d). Addition of 200 μg/mL XOs to the culture, reduced the luminescence counts of 10 ng/ml LPS activated THP-1 cells from 1097 ± 64 to 762 ± 71 (*n* = 4, *p* = 0.0132). Addition of 200 μg/mL XOs to the culture, reduced the luminescence counts of 100 ng/ml LPS activated THP-1 cells from 857 ± 112 to 336 ± 32 (*n* = 4, *p* = 0.0042). Surprisingly, XOs influenced the NFκB activation of non-activated THP-1 cells. Addition of 20 μg/mL XOs upregulated the NFκB activity in THP-1 cells from 109 ± 17 to 203 ± 20 (*n* = 4, *p* = 0.0117). Furthermore, addition of 200 μg/mL XOs upregulated the NFκB activity in THP-1 cells from 109 ± 17 to 215 ± 23 (*n* = 4, *p* = 0.0105). These results suggest that XOs could upregulate or downregulate the NFκB activity in macrophages, which partly recapitulates their parental MSCs, as MSCs themselves have been shown to regulate NFκB^93^. NFκB controls multiple aspects of innate and adaptive immunity, and plays a critical role in regulating the function, activation, and survival of innate immunocytes and inflammatory T cells^94^. NFκB pathway has been reported in response to PDMS^95^, poly(ethylene glycol)^36^, and alginate^37,62^, and reduction in NFκB has been correlated with reduced fibrosis^39,95^.

## Discussion

FBR against implanted materials creates patients discomfort and a variety of health complications^21–23^. Moreover, if the goal is cell transplantation inside a biomaterial, FBR causes a non-functional graft engulfed in a scarring tissue^17^. This is one of the major challenges in clinical translation of tissue engineering and prosthesis products, sensors, and functional cell transplantation. One example is the alginate microcapsules, which has been under research for around 40 years^11^. Some efforts over the past decade have determined that the FBR could be adjusted by purity of alginate^96^, microcapsules size^64^, surface chemistry^24,27^, and alginate composition^18^. We recently showed that even ultrapure alginate activates murine macrophages to secrete pro-inflammatory cytokines, and conditioned media secreted from UC-MSCs suppress such stimulation, partly through interfering with NFκB pathway^62^. Such cytokine secretion is not exclusive to alginate stimulation. Even human macrophages co-cultured with endotoxin-free chitosan or poly(lactic acid) have reported to secrete IL-8, MIP-1, MCP-1, and RANTES or IL-6, IL-8, and MCP-1^97^. Two mechanisms have been described to explain the reasons for alginate-based inflammatory response. Some studies have suggested the presence of immunogens within alginate (such as lipopolysaccharide (LPS), lipoteichoic acid, and peptidoglycans) are the main inducers of inflammation^60,96^. Others reported that such contaminations were undetectable in their alginate^16,24^, and linked the inflammatory response to the inherent properties of alginate. Alginate is a natural acidic polysaccharide extracted from marine brown seaweeds^63,98^. It is composed of different blocks of β-(1, 4)-D-mannuronate (M) and its C5 epimer α-(1, 4)-L-guluronate (G), and Guluronate oligosaccharide derived from alginate has been reported to readily activate macrophages partly through Toll-like receptor 4 (TLR4) signaling pathway^63,98^.

To this end, it can be claimed that an alginate formulation that lacks the inflammatory response could enhance its performance (e.g., functionality of the cell transplants) in immunocompetent rodents. Here we developed a hybrid platform of alginate that could release umbilical cord-derived MSC exosomes in a controlled manner. This platform reduces the inflammatory response against the xenotransplants, leading to >170 days glycemic control in the immunocompetent mouse model of T1D. Even single injection of XOs at the transplantation time delayed the graft rejection for ~40 days on average. Resolving the inflammatory response to transplants have been demonstrated to extend the functional islet transplantation up to a year^1,18,26^. The longevity and mechanism of local immunosuppression have been found to be among the key factors in determining the durability of transplanted islets.

To better understand the therapeutic mechanism of XOs on cellular level, we found that XOs immunosuppressive activity is more pronounce in the heterogenous population of splenocytes compared to when only CD3+ T cells present in the co-cultures (Fig. 4). While the effect of XOs on activated T cells is still debated^91,99^, the present study suggests that XOs are likely to play their immunosuppressive roles through interacting with myeloid lineage. This type of suppression has been widely reported to be due to induction of Tregs^43,58,91^, cell cycle arrest^100^, and adenosinergic immunosuppresion^101^. Interfering with multiple signaling pathways not only makes XOs an exciting therapeutical biologic, but it also suggests potential multi-factorial side effects that could arise from such characteristic. Detailed mechanistical studies in the future need to address the therapeutic vs. side effect aspects of MSC-derived XOs in addition to the questions around their batch-to-batch variations and challenges associated with their storage.

While here the application of AlgXO is focused on the islet transplantation, its core technology could be broadly applicable to other areas of cells transplantation and implants rejection due to immune response.

## Methods

### Isolation and characterization of UC-MSCs and their XOs

Healthy pregnant women at full-term gestation (>37 weeks), maternal age 18–40 years old, and who gave birth at UCI Medical Center were chosen to be used for umbilical cord collection under IRB exemption #2016-2791. Any known complicated pregnancies were excluded from the collection. Umbilical cord-derived mesenchymal stem cells (UC-MSCs) were isolated according to the previously published method with some modifications^102^. Briefly, UCs were washed with PBS under a sterile laminar flow cell culture hood and were cut longitudinally to remove blood vessels. Tissues were then cut into 2–3-mm^3^ segments and incubated with 0.09% collagenase Type II (Sigma) for 45 min at 37 °C in a humidified incubator with 5% CO_2_. After digestion, tissues were passed through 100-μm mesh-sized filters. Cells were then centrifuged at 300 × *g* and 4 °C for 20 min and resuspended in DMEM/F12 (Gibco) supplemented with 10% FBS, 1% penicillin/streptomycin, and 1% L-glutamine. Cells transferred to 175-cm^2^ flasks and incubated at 37 °C in a humidified atmosphere with 5% CO_2_. Flasks were left undisturbed for 2–3 days, after which the medium was changed to remove non-adherent cells. Adherent cells were then characterized for surface markers to further confirm their MSC origin. Supplementary Fig. 1a shows that isolated cells have low expression of Stro-1, high expression CD90/Thy1, CD146/MCAM, CD105/Endoglin, CD166, CD44 while cells are negative for CD19, CD45, and CD106. Such expression profiles are consistent with previous reports^103,104^. UC-MSCs were further cultured in serum-free media for 2 days. Next, exosome isolation was performed as based on reported methods^43,105^. Briefly, conditioned media from cultures of MSC were centrifuged at 300 × *g* for 10 min. Supernatant was collected and transferred to ultracentrifuge tubes (Polyallomer Quick-Seal centrifuge tubes 25 × 89 mm, Beckman Coulter). Samples were then centrifuged in a Beckman Coulter ultracentrifuge (Optima L-90 K or Optima XE- 90 Ultracentrifuge, Beckman Coulter) for 20 min at 16,500 × *g* (Type Ti 45, Beckman Coulter), to remove microvesicles. Supernatant was then carefully collected and centrifuged for 2.5 h with a Type 45 Ti rotor at 4 °C at 120,000 × *g*. Exosome pellet was resuspended in PBS and washed 1X at 4 °C at 120,000 × *g*. The pellet was then resuspended in PBS and stored at −80 °C. XOs were characterized according to an established protocol by International Society of Extracellular Vesicles, where CD63, TSG101, GAPDH, Galectin-1, and Hsp70 were present while and endoplasmic reticulum marker, Calnexin, was absent (Supplementary Fig. 1b). Twenty microliters of XO was mixed with 1X RIPA (Cell Signaling Technologies, USA) buffer and sonicated for 5 min, three times, with vortexing in between. Protein contents were measured using a BCA protein assay kit (Thermo Scientific Pierce, Rockford, IL, USA). Then, 25 μL of BSA standard or 25 μL of sample were transferred to a 96-well plate, and 200 ml working reagent was added. The plate was incubated for 30 min at 37 °C and absorbance was analyzed with a SpectraMax 384 Plus spectrophotometer at 562 nm and the SoftMax Pro software (Molecular Devices, 1311 Orleans Drive, Sunnyvale, CA, USA). Twenty micrograms of protein was then subjected to electrophoresis on a gradient precast polyacrylamide gel (Mini-PROTEAN®; Bio-Rad Laboratories, Hercules, CA, USA). Samples were then transferred onto a nitrocellulose membrane which was then blocked with 5% Blotting Grade Blocker Non-Fat Dry Milk (Bio-Rad Laboratories) in Tris-buffer saline supplemented with %0.1 Polysorbate 20 (TBST) at 4 °C overnight. Membrane was washed with TBST following by incubation with primary antibodies against Calnexin (clone H-70; Santa Cruz Biotechnology, Santa Cruz, CA, USA), Galectin-1/LGALS1 (D608T), Rabbit mAb (Cat# 12936), CD63 Rabbit mAb (Cat# EXOAB-CD63A-1), GAPDH Rabbit mAb (Cat# ab181602), Hsp70 Rabbit mAb (Cat# EXOAB-Hsp70A-1), and TSG101 (clone 4A10; Abcam, Cambridge, UK) dissolved in 0.25% Blotting Grade Blocker Non-Fat Dry Milk in TBST overnight at 4 °C. Next, membrane was washed with TBST for 10 min, in triplicate. Secondary antibodies ECL anti-rabbit IgG horseradish peroxidase-linked F(ab’)2 fragment (donkey, anti-rabbit) (GE Healthcare, Buckinghamshire, UK) were diluted in 0.25% Blotting Grade Blocker Non-Fat Dry Milk in TBST and incubated for 1.5 h. Membranes were analyzed with ECL Prime Western Blotting Detection (GE Healthcare) and a VersaDoc 4000 MP (Bio-Rad Laboratories). XOs were labeled with anti-CD63-modified magnetic beads (Exosome Isolation CD63, Lot OK527, Life Technologies AS, Oslo, Norway) overnight with gentle agitation. The beads were washed with 1% exosome-depleted FBS in PBS and then incubated with human IgG (Sigma-Aldrich) for 15 min at 4 °C. Following another washing step, the beads were incubated with PE-TGFβ, PE/Cy7-PD-L1, and APC/Cy7-MHCII or Isotype Controls (Biolegend, San Diego, USA) for 40 minutes with gentle agitation at room temperature. After another washing step, the samples were analyzed using a FACSAria (BD Bioscience) and data were processed using FlowJo Software (Tri Star, Ashland, OR, USA). Flow cytometry analysis of TGFβ, PD-L1, and MHCII expression on XOs bound to anti-CD63-coated beads demonstrated minimal expression of TGFβ-1 and MHCII, and the absence of PD-L1 (Supplementary Fig. 1d).

### Microcapsules preparation

UPLVG alginate (NovaMatrix^®^, Sandvika, Norway) was fabricated by dissolving 2.5% w/v in 0.9% sterile saline solution and mounted on an air-driven electrostatic microcapsule generator (Nisco Engineering Inc., Oslo, Norway). The alginate solution was added dropwise into a sterile filtered (0.22 μm) gelling solution composed of sterile 20 mM barium chloride and 25 mM HEPES solution to generate circular microcapsules of ~350 microns in diameter. AlgXO microgels were prepared by addition of 7.05 × 10^10^ ± 3.69 × 10^10^ XOs/mL, thawed at RT for 10 to 20 min. Microcapsules were then washed via centrifugation at 100 × *g* and 4 °C for 5 min.

### Dissolving microcapsules and XOs collection

We first optimized microcapsule dissolution using EDTA chelator. EDTA (Sigma‐Aldrich) with concentration of 0.5 M was dissolved in DI water to make a stock solution. Dilutions were performed to test chelator activity at concentrations of 5 and 10 mM from the stock solution. One milliliter of each chelation solution was added on AlgXO or CTRL microcapsules (*n* = 1000 microcapsules/group) and were tested under phase-contrast imaging. Images were obtained using EVOS Imaging system microscope (20/40 PH 2×; ThermoFisher Scientific), and images were captured at 1-min intervals. To determine the dissociation, images were taken of images were analyzed using a microcapsule analysis program (Microcapsule Analysis Program.v5.0.2) in ImageJ. Images were then quantified by the number of microcapsules detected by the program as previously described^106^. Curves were made based on the following percentage: % of dissolved microcapsules = $\frac{{\rm{Detected}}\; {\rm{microcapsules}}\; {\rm{at}}\; t > 0}{{\rm{Detected}}\; {\rm{microacapsules}}\; {\rm{at}}\; t=0}$. Based on the results, microcapsules were dissolved for 10 min in 10 mM EDTA solution (Supplementary Fig. 3). To quantify the XOs encapsulated within AlgXO, dissolved solution was then subjected to ultracentrifugation for 2.5 h at 4 °C and 120,000 × *g*. Exosome pellet was resuspended in PBS and stored at −80 °C until further analyses.

### Rat islet quality control and viability

Four- to six-week-old male Sprague-Dawley rats (Envigo Harlan, Houston, TX) were used as islet donors. Islet isolation was performed using standard collagenase digestion and gradient purification. Common duct was clamped on the side of mesoduodenum. Ice-cold collagenase V solution (6–7 ml with concentration of 1 mg/ml in HBSS+) was injected into common bile duct (CBD) using 23G needle. Pancreas was then removed from dorsal wall of the abdominal cavity and transferred into 50 ml conical tube on ice box. Pancreas in the conical tubes were kept at 37 ˚C water bath (with 30 rpm shaking) for 17 min, after which 20 ml cold HBSS+ was added into the conical tube and were hand-shaken strongly. Islets were then isolated and purified using non-continuous density gradient. From each isolated batch, islets were tested for their quality including DTZ, viability, and glucose-stimulated insulin release (GSIR) assay were conducted. Upon each batch of islet isolation and prior to implantation, we ran quality control tests to identify the suitability of islets viability and function for transplantation. Islet count and purity (per rat pancreas) was 947 ± 137 IEQ as measured using DTZ staining (Supplementary Fig. 4). Viability of each isolation batch was more than 90%, and on average, it was 93% ± 2%. To quantify the viability of islets, 100 IEQ islets (either encapsulated or naked) were stained with Calcein AM (CalAM, Invitrogen, Cat# C1430) for live cells and propidium iodide (PI, Invitrogen, Cat# P3566) for dead and dying cells for 30 min. Stained islets were analyzed using a microplate reader (Tecan Infinite F200; Tecan). The islet viability was calculated by the equation: (CalAM^+^ cells)/(CalAM^+^ cells + PI^+^ cells) × 100. GSIR assay was conducted to assure the islet quality prior to implantation. From each isolation batch, three technical replicates of 100 IE islets per sample were incubated at 37 °C and 5% CO_2_ for 1 h in each media in the corresponding order: low glucose (2.8 mmol/L; L1), high glucose (28 mmol/L; H), high glucose plus 3‐isobutyl‐1‐methylxanthine (28 mmol/L + 0.1 mmol/L IBMX; H+), and last back to low glucose (2.8 mmol/L; L2). Supernatant was collected and stored at −20 °C until analysis. Insulin concentration released during incubation was measured using a porcine insulin enzyme‐linked immunosorbent assay (Mercodia, cat#10‐1200‐01). Absorbance was then measured using a microplate reader with 450‐nm wavelength filter (Tecan Infinite F200 and Magellan V7) and presented as (μg/L). Stimulation index (SI) was calculated as the ratio of insulin concentration secreted in high glucose over the insulin concentration secreted in the first low‐glucose incubation. Our islet quality control criteria were: SI units >2, Viabilities >90%, and purities >90% (DTZ).

### Scanning electron microscopy

Both AlgXO or CTRL microcapsules were air-dried in an sterile chamber prior to SEM analysis. Dried samples were placed on carbon-tapped imaging stubs. We used Philips XL-30 FEG SEM with EDS (Noran 6) system, which is a thermionic field emission SEM with a fully automatic gun configuration controlled by advanced computer technology (the magnification is up to ×800,000 with 2-nm resolution). The working distance was adjusted to be 10 mm at 0.5 kV voltage, and 10 pA as the beam current.

### Streptozotocin injection in mice

C57/BL6 mice were fasted overnight (at least 12 h) prior to Streptozotocin (STZ; Sigma CAS#: 18883-66-4) injection. STZ (180 mg/kg of mice body weight) was dissolved in 10 ml STZ buffer (0.1 M Sodium Citrate buffer pH = 4.5) before injection. The buffer was vortexed and kept on ice for about 15 min prior to i.p. administration. To assure the STZ induction, mice had to be hyperglycemic for at least a week, and defined as non-fasting blood glucose levels ≥350 mg/dl from the tail vein. To minimize the surgery-induced mortalities, mice blood glucose was adjusted prior to transplantation via insulin injection. All the blood glucose reads in this study are non-fasting.

### Islet transplantation

Animal surgeries and protocols were carried out in compliance with all relevant ethical regulations, as approved by the UCI Committee on Animal Care (IACUC). STZ-induced diabetic or non-diabetic immune-competent (male C57BL/6 mice; Jackson Laboratory) with 8- to 10-week-age were anesthetized with 2.5% isoflurane, and then their abdomens (or top-backs) shaved and sterilized using betadine and 70% ethanol. Injections using 1 mL pipet were used for microcapsules (either with or without islets) transplantation into a 0.5 cm incision that was made along the top-back for implantation. For intraperitoneal transplantation, a 0.5–1.0 cm incision was created along the abdomen midline and the peritoneal wall followed by exposure to blunt dissection. Microcapsules were loaded into sterile pipette tip for injection. Then the peritoneal wall was closed with sutures.

### Glucose tolerance testing

Mice were fasted 10–14 h prior to oral glucose tolerance testing (OGTT) measurements. Next, a fresh glucose solution was prepared by dissolving 30% glucose in DPBS (3 mg/kg of mice body weight). Prior to glucose administration, mice blood glucose was measure. Mice were anesthetized with 2% isoflurane inhalation, and the glucose solution was orally injected by oral gavage. Next, blood glucoses were measured through tail-vein snipping upon 10, 20, 30, 60, 90, 120, and 180 min after glucose injection. Blood samples obtained from the tail vein were measured for glucose levels using a glucometer (CONTOUR®NEXT glucometer, Ascensia Diabetes Care, Parsippany, NJ).

### Fibrotic tissue sectioning

Fibrotic tissues (containing microcapsules) were cut and fixed in 4% PFA at 4 °C overnight. Next, tissues were washed with PBS 3× and embedded in 2% agar (CAT#: A1296, Sigma, USA). Agar molds were then embedded in plastic with wax. The entire cassette was placed in 58 °C paraffin bath for 15 min. Tissues were then sectioned with 7-μm thickness using an RM2255 microtome (Leica) with Superfrost slides. Prior to staining, an ethanol gradient dehydration and paraffin embedding cycle were performed.

### Lavage and fibrotic tissue flow cytometry

Prior to removing the implants from the subcutaneous or intraperitoneal areas, small incision was created on the distant site from the explants. One milliliter of cold DPBS was injected back and forth for 3× with pipette around the fibrotic microcapsules and suspended cells were removed and washed with DPBS. In the case of cytokine analysis, the lavage collected from intraperitoneal cavity was immediately frozen at -80 °C freezer until being shipped on dry ice to Eve Technologies (Calgary, Canada), where cytokines were analyzed using Mouse Focused 32-Plex Discovery Assay (CAT#: 17619). To analyze the cell populations, isolated cells were stained with CD3 (1:500 dilution, Biolegend Cat#: 100203), CD11b (1:200 dilution, Biolegend Cat#: 101211), I-A/I–E (1:200 dilution, Biolegend Cat#: 107628) CD19 (1:200 dilution, Biolegend Cat#: 115507) and CD206 (1:200 dilution, Biolegend, Cat#: 141711) in 2% BSA and 1% heat-inactivated FBS. Similar panel was used for the cells isolated from fibrotic tissues around microcapsules with a slight difference. To isolate cells from fibrotic tissues, they were first minced into 2–5 mm pieces and then microcapsules were dissolved using 10 mM EDTA (see Supplementary Fig. 3). Clustering of flow cytometry data was completed by concatenating all 3 biological replicates into one file, and clustering with the tSNE (t-distributed stochastic neighbor embedding) plugin for 1000 iterations, operating at theta = 0.5. Data are displayed as user-gated populations graphed against their respective X and Y tSNE coordinates.

### Click-iT Plus TUNEL assay

To analyze the viability of transplanted islets in vivo, we explanted microcapsules 1 month after transplantation, and conducted the TUNEL assay according to the manufacturer protocol (CAT#: C10617, Invitrogen). Briefly, microcapsules were washed 3× with ice-cold PBS and fixed in 4% formalin for 24 h at 4 °C. Samples were permeabilized using 1× RIPA buffer for 20 min and rinsed with ice-cold PBS. TdT reaction was performed following by the Click-iT Plus reaction. Finally, DAPI counterstaining was conducted by 1:2000 dilution for 15 min, and microcapsules were images using Olympus FV3000 Laser-Scanning Confocal Spectral Inverted Microscope (Olympus, USA). Total signal area was then quantified using imageJ analyses, and area percentages were compared for islets in both AlgXO vs CTRL microcapsules.

### Controlled-release studies

After fabrication, ~1000 AlgXO and CTRL microcapsules were plated in a 6-well plate at 37 °C in a humidified incubator with 5% CO_2_. At indicated timepoints after co-incubation (Fig. 3g), 1 mL of the culture supernatant was collected and 1 mL of sterile DPBS was replaced inside the well to keep the culture volume constant. Plates were sealed to minimize the loss of water due to evaporation. Isolated media was then measured for total protein concentration, and exosomal content using NTA.

### Nanoparticle tracking analysis

NTA was performed using the Nanosight NS3000system (Malvern Instruments, USA). XOs (either from ultracentrifugation process or controlled-release experiment) were suspended in PBS to contain ~107–1010 particles per ml, which fits within the detection limits of Nanosight NS3000. Exosomes were analyzed based on light scattering using an optical microscope aligned perpendicularly to the beam axis. A 60-s video was recorded and subsequently analyzed using NTA software.

### Captive bubble contact angle

We used a custom-made captive bubble, modifying the regular contact angel equipment (MCA-3, Kyowa Interface Science). Thin AlgXO and CTRL hydrogels were formed within a capillary space between two glass slides. Hydrogels on top of the glass slides were then merged into water beaker, and camera was focused on the hydrogel. Small air bubbles were then shut on the surface of hydrogel, creating the aqueous–solid–gas phase on the hydrogels surface. Images captured from the bubbles and contact angles were measured using ImageJ software with contact angle plugin, using circular and/or elliptical fits wherever appropriate.

### Microcapsule mechanical and physical properties

Mechanical properties of microcapsules were measured using a microscale tension-compression test system (MicroTester G2, CellScale, Ontario, Canada). The probe was constructed by attaching a 1 mm × 1 mm platen to a 154 μm cantilever and mounted to the instrument. Microcapsules were transferred by pipette into the test chamber, which was pre-filled with water. Single microcapsules were isolated using the platen-cantilever set-up, oriented by the attached microscope on the MicroTester to be in focus. The force as a function of time was measured for compressive strains of 0–50% using a 200 s loading time, a 10 s hold time, and a 20 s release time. Force resolution was adjusted at 1 μN and spatial resolution at 1.5 µm. Measurements were recorded at 200-ms intervals. The force-displacement data was then converted into stress–strain, with the associated curve used to obtain a linear regression line from the stress–strain curve with <0.2 strain.

### H&E, Masson’s trichrome, and immunofluorescence staining

Trichrome staining was used to visualize collagen fibrosis around capsules. CTRL microcapsules were retrieved from mice after 2 weeks and fixed overnight using 4% paraformaldehyde at 4 °C, following by embedding in paraffin and sectioning. Xylene was used to deparaffinize sections prior to tissue staining. hematoxylin and eosin (H&E) staining was done following the standard procedure, and slides were mounted using Permount (Fisher Scientific) and 0.17-mm glass coverslips. Then, tissue samples were mounted on slides, and imaged under Nikon Ti–E fluorescent Microscope (Leica, USA).

Immunofluorescence imaging was performed to determine immune populations infiltrated around microcapsules. Microcapsules collected after 2 weeks of subcutaneous implantation were then blocked in agar and underwent paraffin embedding process then cut and mounted. Alcohol and xylene processing were performed to deparaffinized the samples then the spheres underwent heat-mediated antigen retrieval in pressure cooker with citrate buffer solution. The microcapsules were then blocked for 1 h using a 1% bovine serum albumin (BSA) solution. Next, tissue slides containing microcapsules were incubated for 1 h in an immunostaining cocktail solution consisting of DAPI (500 nM), αSMA (1:500 dilution, Biolegend Cat#: MMS-466S), CD68 (1:200 dilution, Biolegend Lot#: B229996), CD3 (1:500 dilution, Biolegend Cat#: 100203), CD11b (1:200 dilution, Biolegend Cat#: 101211), I-A/I–E (1:200 dilution, Biolegend Cat#: 107628), and CD206 (1:200 dilution, Biolegend, Cat#: 141711) in 2% BSA. To stain the microcapsules collected from i.p. cavity, they were washed three times with a 0.1% Tween 20 dissolved in 5% BSA solution and maintained in a 50% glycerol solution. Spheres were then transferred to glass slides and imaged using an Olympus FV3000 Laser-Scanning Confocal Spectral Inverted Microscope (Olympus, USA) equipped with 5 and ×10 objectives. 405, 488, and 640 nm solid-state lasers were used, and the laser power was adjusted to be 1–1.5% in all channels.

Protein adsorption was also conducted via co-incubation of IgG fluorescent antibody (PE mouse IgG^1κ^ isotype ctrl clone: MOPC-21, Biolegend, CAT#: 400111, 1:200) with AlgXO or CTRL microcapsules for 24 h on a shaking plate at 37 °C. Microcapsules were then washed 2× with 5 mL PBS and transferred to glass slides and imaged using an Olympus FV3000 Laser-Scanning Confocal Spectral Inverted Microscope (Olympus, USA). The 488 nm solid-state laser was used, and the laser power was adjusted to be 1–1.5%.

### Human PBMCs proliferation and cytokine assay

Peripheral blood mononuclear cells (PBMCs) were isolated from buffy coats from healthy and anonymous blood donors (UCI Institute for Clinical and Transitional Science) by density gradient centrifugation (Ficoll-Pague plus, GE Healthcare). For proliferation assay, 20 μg of XOs were incubated with 1 × 10^5^ CFSE [5(6)-carboxyfluoresceindiacetate N-succinimidyl ester] (Molecular Probes, Eugene, OR) labeled PBMCs. To activate T-cell proliferation, Dynabeads™ Human T-Activator CD3/CD28 for T-cell expansion and activation was used with 1:1 ratio of PBMCs:Dynabeads^TM^. PBMCs proliferation was analyzed after 4 days using flow cytometry (FACSAria, BD) and data were analyzed using the FlowJo. For cytokine analysis, cells were cultured in RPMI 1640 with 10% heat-inactivated FBS, 1% penicillin/streptomycin, and 1% L-glutamine. Cells transferred to 96 or 48 well plates and incubated at 37 °C in a humidified atmosphere with 5% CO_2_. Dynabeads Human T-Activator CD3/CD28 for T-cell expansion and activation was used with 1:1 ratio of PBMCs:Dynabeads. The XOs were mixed with fresh culture media (with 20 and 200 μg/mL concentrations). DynaBeads were then added to isolated PBMCs in the presence and absence of XOs. Supernatants were collected and Luminex assay was used to analyze the secreted cytokines. Fifty microliters of PBMC culture supernatants were collected and either frozen at −80 °C or immediately analyzed using a human custom ProcartaPlex (11plex, ThermoFisher Scientific, Vienna, Austria) with Luminex 77. Results were then reported as mean fluorescence intensity (MFI).

### Splenocytes and T-cells proliferation assay

Spleens from FVB/n mice were purchased from the Jackson laboratory male mice were dissected, filtered into a single-cell suspension using 70 μm sterile filter, and red blood cells were removed using Tris-acetic-acid-chloride (TAC). Splenocytes were washed once with PBS and resuspended at 15 × 10^6^/mL in staining buffer (0.01% BSA in PBS). Splenocytes were stained with proliferation dye eFluor^TM^ 670 (ThermoFisher Scientific, CAT#: 65-0840-85) using 5 mM dye per 10 M cells and incubated in a 37 °C water bath for 10 min. Finally, cells were washed and resuspended at 1 M/mL in RPMI 1640 w/ HEPES + L-glutamine (Gibco, CAT#: 22400-105) complete medium containing 10% FBS (Atlanta Biologicals, CAT#: S11150), 1X non-essential amino acids (Gibco, CAT#: 11146-050), 100 U/mL penicillin–100 µg/mL streptomycin (Gibco, CAT#: 15140163), 1 mM sodium pyruvate (Gibco, CAT#:11360-070), and 55 μM β-mercaptoethanol (Gibco, CAT#:21985-023), eFluor^TM^ 670-labeled Splenocytes were plated (50 × 10^3^/well) in a U-bottom 96-well plate (VWR, CAT#: 10062-902) and activated with plate-bound anti-Armenian hamster IgG (30 µg/mL, Jackson Immuno Research, CAT#:127-005-099) with CD3 (0.5 μg/mL, Tonbo, CAT#: 70-0031) and CD28 (1 μg/mL, Tonbo, CAT#: 70-0281). XOs with 20 or 200 μg/mL concentration were added to the co-cultures after cell seedings. After 4 days of culture, cells were stained with Zombie Live/Dead Dye (BioLegend, CAT#: 423105) and live cells were analyzed for proliferation.

Similar procedure was conducted for T lymphocytes, where isolated splenocytes were subjected to EasySep^TM^ Mouse T cell Isolation Kit (StemCell Technologies, CAT#: 19851) according to the manufacturer’s instructions. After 4 days of co-cultures, T cells were collected and blocked with anti-mouse CD16/32 (BioLegend, CAT#: 101302), stained with Zombie Live/Dead Dye and fluorescent-conjugated antibodies: CD4 (BioLegend, CAT#: 100512; clone RM4-5) and CD8 (BioLegend, CAT#: 100709; clone 53-6.7). Cells were processed using the BD LSR II or BD LSRFortessa^TM^ X-20 flow cytometer and analyzed using FlowJo software v10.0.7 (Tree Star, Inc).

### Macrophage activation assay

RAW 264.7 cells were purchased from ATCC (CAT# TIB-71) and NFκB reporter THP-1_Lucia human cell lines were purchased from InvivoGen (CAT#: thpl-nfkb) employed for downstream experiments of this study. Passages 5–10 were cultured in RPMI 1640 supplemented with 10% of heat-inactivated FBS in the presence of 1% penicillin/streptomycin and 1% L-glutamine. Cells were then stimulated with 10 or 100 ng/mL of LPS (Invitrogen, CAT#: 50-112-2025). Stimulated and non-stimulated cells were then mixed with XOs with the mentioned concentrations in the results section. Control cells, LPS-stimulated cells in the presence and absence of XOs, and non-stimulated cells in the presence and absence of XOs (100,000 cells for each condition) were co-cultured for 10–14 h at 37 °C in a humidified incubator with 5% CO_2_. Next, supernatant was collected for cytokine analyses. Supernatants were centrifuged at 2500 × *g* and 4 °C for 5 min and stored at −80 °C. Samples were then shipped on dry ice to Eve Technologies (Calgary, Canada), where cytokines were analyzed using Mouse Focused 32-Plex Discovery Assay (CAT#: 17619).

### IVIS imaging

NFκB reporter THP-1_Lucia human cell lines were used to measure the NFκB activity. These cells are engineered THP-1 monocyte cell line by stable integration of an NFκB-inducible Luc reporter construct. The levels of NFκB-induced secreted luciferase in the cell culture supernatant are readily assessed with Quanti-Luc (CAT#: rep-qlc2). As a result, these cells could quantitatively measure NFκB activation. Cell were cultured in a phenol-free media and supernatants (as described in the in vitro co-culture section of the “Methods”) were collected. QUANT-Luc assay solution was added with a concentration of 1 mg/mL and incubated for 30 s. The resulted plate was then imaged in an IVIS imager (or VersaDoc 4000 MP). Exposure time was adjusted as 0.2 s, field of view 12.5, f number 16, and binning factor of 4 were selected as optimized acquisition settings.

### Animal studies

All animal procedures were performed under approved University of California Irvine, Institutional Animal Care and Use Committee (Protocol #: AUP-17-241), in accordance with the guidelines of the National Institutes of Health.

### Statistics and reproducibility

Sample numbers were selected based on power analyses in most cases. Statistical analyses and the number of replicates were determined for each assay and are mentioned separately for each figure.

### Reporting summary

Further information on research design is available in the Nature Research Reporting Summary linked to this article.

## Supplementary information

Supplementary Information

Description of Supplementary Files

Supplementary Data 1

Reporting Summary

## Data Availability

All relevant data are available within the article and Supplementary Information, and from the corresponding author upon reasonable request. Source data is given in Supplementary Data 1 file.

## References

[1] Vegas AJ (2016). Long-term glycemic control using polymer-encapsulated human stem cell–derived beta cells in immune-competent mice. Nat. Med..

[2] Mao AS (2019). Programmable microencapsulation for enhanced mesenchymal stem cell persistence and immunomodulation. Proc. Natl Acad. Sci. USA.

[3] Kojima R (2018). Designer exosomes produced by implanted cells intracerebrally deliver therapeutic cargo for Parkinson’s disease treatment. Nat. Commun..

[4] Parmar M, Grealish S, Henchcliffe C (2020). The future of stem cell therapies for Parkinson disease. Nat. Rev. Neurosci..

[5] Wehling M (2014). Non-steroidal anti-inflammatory drug use in chronic pain conditions with special emphasis on the elderly and patients with relevant comorbidities: management and mitigation of risks and adverse effects. Eur. J. Clin. Pharmacol..

[6] Srinivasan A, De Cruz P (2017). Review article: a practical approach to the clinical management of NSAID enteropathy. Scand. J. Gastroenterol..

[7] Tekin Z (2016). Outcomes of pancreatic islet allotransplantation using the edmonton protocol at the University of Chicago. Transpl. Direct.

[8] Shapiro AMJ (2000). Islet transplantation in seven patients with type 1 diabetes mellitus using a glucocorticoid-free immunosuppressive regimen. N. Engl. J. Med..

[9] Shapiro AMJ (2006). International trial of the edmonton protocol for islet transplantation. N. Engl. J. Med..

[10] Desai T, Shea LD (2016). Advances in islet encapsulation technologies. Nat. Rev. Drug Discov..

[11] Franklin Lim, F. & Sun, A. M. Microencapsulated islets as bioartificial endocrine pancreas. *Science* **210**, 908–910 (1980). 10.1126/science.6776628 6776628

[12] Tuch BE (2009). Safety and viability of microencapsulated human islets transplanted into diabetic humans. Diabetes Care.

[13] Basta G (2011). Long-term metabolic and immunological follow-up of nonimmunosuppressed patients with type 1 diabetes treated with microencapsulated islet allografts. Diabetes Care.

[14] Orive, G. et al. Engineering a clinically translatable bioartificial pancreas to treat type I diabetes. *Trend. Biotechnol*. **36**, 445–456 (2018). 10.1016/j.tibtech.2018.01.007 29455936

[15] Evron Y (2018). Long-term viability and function of transplanted islets macroencapsulated at high density are achieved by enhanced oxygen supply. Sci. Rep..

[16] Doloff JC (2017). Colony stimulating factor-1 receptor is a central component of the foreign body response to biomaterial implants in rodents and non-human primates. Nat. Mater..

[17] Bochenek MA (2018). Alginate encapsulation as long-term immune protection of allogeneic pancreatic islet cells transplanted into the omental bursa of macaques. Nat. Biomed. Eng..

[18] Farah S (2019). Long-term implant fibrosis prevention in rodents and non-human primates using crystallized drug formulations. Nat. Mater..

[19] de Vos P, Hamel AF, Tatarkiewicz K (2002). Considerations for successful transplantation of encapsulated pancreatic islets. Diabetologia.

[20] Vaithilingam, V. & Tuch, B. E. Islet transplantation and encapsulation: an update on recent developments. *Rev. Diabet. Stud.* **8**, 51 (2011). 10.1900/RDS.2011.8.51 PMC3143677 21720673

[21] Mohammadi, M. R., Luong, J. C., Kim, G. G., Lau, H. & Lakey, J. R. T. in *Handbook of Tissue Engineering Scaffolds*, Vol. 1 (eds Mozafari, M., Sefat, F. & Atala, A.) (Woodhead Publishing, 2019).

[22] Swanson, E. Analysis of US Food and Drug Administration breast implant postapproval studies finding an increased risk of diseases and cancer: why the conclusions are unreliable. *Ann. Plast. Surg*. **82**, 253–254 (2019). 10.1097/SAP.0000000000001732 PMC6392206 30730863

[23] Headon H, Kasem A, Mokbel K (2015). Capsular contracture after breast augmentation: an update for clinical practice. Arch. Plast. Surg..

[24] Vegas AJ (2016). Combinatorial hydrogel library enables identification of materials that mitigate the foreign body response in primates. Nat. Biotechnol..

[25] Headen DM (2018). Local immunomodulation with Fas ligand-engineered biomaterials achieves allogeneic islet graft acceptance. Nat. Mater..

[26] Alagpulinsa DA (2019). Alginate-microencapsulation of human stem cell-derived β cells with CXCL12 prolongs their survival and function in immunocompetent mice without systemic immunosuppression. Am. J. Transplant..

[27] Liu Q (2019). Zwitterionically modified alginates mitigate cellular overgrowth for cell encapsulation. Nat. Commun..

[28] Spasojevic, M. et al. Reduction of the inflammatory responses against alginate-poly-L-lysine microcapsules by anti-biofouling surfaces of PEG-b-PLL diblock copolymers. *PLoS ONE* **9**, e109837 (2014). 10.1371/journal.pone.0109837 PMC4209974 25347191

[29] Vacanti NM (2012). Localized delivery of dexamethasone from electrospun fibers reduces the foreign body response. Biomacromolecules.

[30] Hachim D, LoPresti ST, Yates CC, Brown BN (2017). Shifts in macrophage phenotype at the biomaterial interface via IL-4 eluting coatings are associated with improved implant integration. Biomaterials.

[31] Cannarile, M. A. et al. Colony-stimulating factor 1 receptor (CSF1R) inhibitors in cancer therapy. J. Immunother. *Cancer* **5**, 53 (2017). 10.1186/s40425-017-0257-y PMC5514481 28716061

[32] Papadopoulos KP (2017). First-in-human study of AMG 820, a monoclonal anti-colony-stimulating factor 1 receptor antibody, in patients with advanced solid tumors. Clin. Cancer Res..

[33] Sanchez AB (2016). CXCL12-induced neurotoxicity critically depends on NMDA receptor-gated and l-type Ca^2+^ channels upstream of p38 MAPK. J. Neuroinflammation.

[34] Lin JT (2008). TNFα blockade in human diseases: an overview of efficacy and safety. Clin. Immunol..

[35] Walton KL, Johnson KE, Harrison CA (2017). Targeting TGF-β mediated SMAD signaling for the prevention of fibrosis. Front Pharm..

[36] Amer, L. D. et al. Inflammation via myeloid differentiation primary response gene 88 signaling mediates the fibrotic response to implantable synthetic poly(ethylene glycol) hydrogels. *Acta Biomater*. **100**, 105–117 (2019). 10.1016/j.actbio.2019.09.043 PMC6980661 31568879

[37] Yang D, Jones KS (2009). Effect of alginate on innate immune activation of macrophages. J. Biomed. Mater. Res. Part A.

[38] Lawlor C (2016). Treatment of *Mycobacterium tuberculosis*-infected macrophages with poly(lactic-co-glycolic acid) microparticles drives NFκB and autophagy dependent bacillary killing. PLoS ONE.

[39] Moore, L. B. & Kyriakides, T. R. in *Immune Responses to Biosurfaces* (eds Lambris, J. D., Ekdahl, K. N., Ricklin, D. & Nilsson, B.) (Springer International Publishing, 2015).

[40] Su VY-F, Lin C-S, Hung S-C, Yang K-Y (2019). Mesenchymal stem cell-conditioned medium induces neutrophil apoptosis associated with inhibition of the NF-κB pathway in endotoxin-induced acute lung injury. Int. J. Mol. Sci..

[41] Vigo T (2017). IFN-γ orchestrates mesenchymal stem cell plasticity through the signal transducer and activator of transcription 1 and 3 and mammalian target of rapamycin pathways. J. Allergy Clin. Immunol..

[42] Chen, C.-P., Tsai, P.-S.Huang, C.-J. (2012). Antiinflammation effect of human placental multipotent mesenchymal stromal cells is mediated by prostaglandin E2 via a myeloid differentiation primary response gene 88-dependent pathway. Anesthesiology.

[43] Riazifar M (2019). Stem cell-derived exosomes as nanotherapeutics for autoimmune and neurodegenerative disorders. ACS Nano.

[44] Yin JQ, Zhu J, Ankrum JA (2019). Manufacturing of primed mesenchymal stromal cells for therapy. Nat. Biomed. Eng..

[45] Riazifar M, Pone EJ, Lötvall J, Zhao W (2017). Stem cell extracellular vesicles: extended messages of regeneration. Annu. Rev. Pharmacol. Toxicol..

[46] Lankford KL (2018). Intravenously delivered mesenchymal stem cell-derived exosomes target M2-type macrophages in the injured spinal cord. PLoS ONE.

[47] Fan Y (2018). Human fetal liver mesenchymal stem cell-derived exosomes impair natural killer cell function. Stem Cells Dev..

[48] Burrello J (2016). Stem cell-derived extracellular vesicles and immune-modulation. Front. Cell Develop. Biol..

[49] Khare D (2018). Mesenchymal stromal cell-derived exosomes affect mRNA expression and function of B-lymphocytes. Front. Immunol..

[50] Carreras-Planella L, Monguió-Tortajada M, Borràs FE, Franquesa M (2019). Immunomodulatory effect of MSC on B cells is independent of secreted extracellular vesicles. Front. Immunol..

[51] Shigemoto-Kuroda T (2017). MSC-derived extracellular vesicles attenuate immune responses in two autoimmune murine models: type 1 diabetes and uveoretinitis. Stem Cell Rep..

[52] Hass R, Kasper C, Böhm S, Jacobs R (2011). Different populations and sources of human mesenchymal stem cells (MSC): a comparison of adult and neonatal tissue-derived MSC. Cell Commun. Signal.

[53] Sun Y (2018). Human mesenchymal stem cell derived exosomes alleviate type 2 diabetes mellitus by reversing peripheral insulin resistance and relieving β-cell destruction. ACS Nano.

[54] Matsumoto S (2014). Clinical porcine islet xenotransplantation under comprehensive regulation. Transplant. Proc..

[55] Ekser B, Bottino R, Cooper DKC (2016). Clinical islet xenotransplantation: a step forward. EBioMedicine.

[56] Rezaa Mohammadi M, Rodrigez S, Cao R, Alexander M, Lakey JRT (2018). Immune response to subcutaneous implants of alginate microcapsules. Mater. Today.: Proc..

[57] Nie W (2018). Human mesenchymal-stem-cells-derived exosomes are important in enhancing porcine islet resistance to hypoxia. Xenotransplantation.

[58] Zhang B (2018). Mesenchymal stromal cell exosome-enhanced regulatory T-cell production through an antigen-presenting cell-mediated pathway. Cytotherapy.

[59] Bai L (2017). Effects of mesenchymal stem cell-derived exosomes on experimental autoimmune uveitis. Sci. Rep..

[60] Paredes-Juarez GA, de Haan BJ, Faas MM, de Vos P (2013). The role of pathogen-associated molecular patterns in inflammatory responses against alginate based microcapsules. J. Control. Release.

[61] Paredes Juárez, G. A., Spasojevic, M., Faas, M. M. & de Vos, P. Immunological and technical considerations in application of alginate-based microencapsulation systems. *Front. Bioeng. Biotechnol.* **2**, 26 (2014). 10.3389/fbioe.2014.00026 PMC4123607 25147785

[62] Mohammadi, M. et al. Controlled release of stem cell secretome attenuates inflammatory response against implanted biomaterials. *Adv. Healthc. Mater.* **9**, e1901874 (2020). 10.1002/adhm.201901874 32419390

[63] Fang W (2017). Identification and activation of TLR4-mediated signalling pathways by alginate-derived guluronate oligosaccharide in RAW264.7 macrophages. Sci. Rep..

[64] Veiseh O (2015). Size- and shape-dependent foreign body immune response to materials implanted in rodents and non-human primates. Nat. Mater..

[65] Shi C, Pamer EG (2011). Monocyte recruitment during infection and inflammation. Nat. Rev. Immunol..

[66] Madan R (2014). Role of leptin-mediated colonic inflammation in defense against *Clostridium difficile* colitis. Infect. Immun..

[67] Lacey DC (2012). Defining GM-CSF- and macrophage-CSF-dependent macrophage responses by in vitro models. J. Immunol..

[68] Yoshimura T (2018). The chemokine MCP-1 (CCL2) in the host interaction with cancer: a foe or ally?. Cell. Mol. Immunol..

[69] Chung, L. et al. Interleukin 17 and senescent cells regulate the foreign body response to synthetic material implants in mice and humans. *Sci. Transl. Med*. **12**, eaax3799 (2020). 10.1126/scitranslmed.aax3799 PMC7219543 32295900

[70] Seong S-Y, Matzinger P (2004). Hydrophobicity: an ancient damage-associated molecular pattern that initiates innate immune responses. Nat. Rev. Immunol..

[71] Yesilyurt V (2017). A facile and versatile method to endow biomaterial devices with zwitterionic surface coatings. Adv. Healthc. Mater..

[72] Anderson JM, Rodriguez A, Chang DT (2008). Foreign body reaction to biomaterials. Semin. Immunol..

[73] Hu WJ, Eaton JW, Tang L (2001). Molecular basis of biomaterial-mediated foreign body reactions. Blood.

[74] Eslami-Kaliji F, Sarafbidabad M, Rajadas J, Mohammadi MR (2020). Dendritic cells as targets for biomaterial-based immunomodulation. ACS Biomater. Sci. Eng..

[75] Jain N, Vogel V (2018). Spatial confinement downsizes the inflammatory response of macrophages. Nat. Mater..

[76] Meli VS (2019). Biophysical regulation of macrophages in health and disease. J. Leukoc. Biol..

[77] Pacienza N (2019). In vitro macrophage assay predicts the in vivo anti-inflammatory potential of exosomes from human mesenchymal stromal cells. Mol. Ther. Methods Clin. Dev..

[78] Lenzini, S., Bargi, R., Chung, G. & Shin, J.-W. Matrix mechanics and water permeation regulate extracellular vesicle transport. *Nat. Nanotechnol.* **15**, 217–223 (2020). 10.1038/s41565-020-0636-2 PMC7075670 32066904

[79] Zhang B (2014). Mesenchymal stem cells secrete immunologically active exosomes. Stem Cells Dev..

[80] de Witte SFH (2018). Immunomodulation by therapeutic mesenchymal stromal cells (MSC) is triggered through phagocytosis of MSC by monocytic cells. Stem Cells.

[81] Guha M, Mackman N (2001). LPS induction of gene expression in human monocytes. Cell. Signal..

[82] Sommerfeld SD (2019). Interleukin-36γ–producing macrophages drive IL-17–mediated fibrosis. Science. Immunology.

[83] Luckheeram, R. V., Zhou, R., Verma, A. D. & Xia, B. CD4+ T cells: differentiation and functions. *J. Immun. Res.* **2012**, 10.1155/2012/925135 (2012). 10.1155/2012/925135 PMC3312336 22474485

[84] Acharya S (2018). Amelioration of Experimental autoimmune encephalomyelitis and DSS induced colitis by NTG-A-009 through the inhibition of Th1 and Th17 cells differentiation. Sci. Rep..

[85] Croft M (2009). The role of TNF superfamily members in T-cell function and diseases. Nat. Rev. Immunol..

[86] Korn T (2008). IL-6 controls Th17 immunity in vivo by inhibiting the conversion of conventional T cells into Foxp3 regulatory T cells. Proc. Natl Acad. Sci. USA.

[87] Lee Y (2012). Induction and molecular signature of pathogenic TH17 cells. Nat. Immunol..

[88] Tasso R (2008). Development of sarcomas in mice implanted with mesenchymal stem cells seeded onto bioscaffolds. Carcinogenesis.

[89] Tasso R (2012). Mesenchymal stem cells induce functionally active T-regulatory lymphocytes in a paracrine fashion and ameliorate experimental autoimmune uveitis. Investigative Ophthalmol. Vis. Sci..

[90] English K (2009). Cell contact, prostaglandin E2 and transforming growth factor beta 1 play non-redundant roles in human mesenchymal stem cell induction of CD4+CD25^High^forkhead box P3+ regulatory T cells. Clin. Exp. Immunol..

[91] Zhang B (2013). Mesenchymal stem cells secrete immunologically active exosomes. Stem Cells Dev..

[92] Mansouri, N. et al. Mesenchymal stromal cell exosomes prevent and revert experimental pulmonary fibrosis through modulation of monocyte phenotypes. *JCI Insight* **4**, e128060 (2019). 10.1172/jci.insight.128060 PMC6948760 31581150

[93] Capcha JMC (2019). Wharton’s jelly-derived mesenchymal stem cells attenuate sepsis-induced organ injury partially via cholinergic anti-inflammatory pathway activation. Am. J. Physiol.-Regulatory, Integr. Comp. Physiol..

[94] Liu T, Zhang L, Joo D, Sun S-C (2017). NF-κB signaling in inflammation. Signal Transduct. Target. Ther..

[95] Moore, L. B., Sawyer, A. J., Charokopos, A., Skokos, E. A. & Kyriakides, T. R. Loss of monocyte chemoattractant protein-1 alters macrophage polarization and reduces NFkappaB activation in the foreign body response. *Acta Biomater.* **11**, 37–47 (2015). 10.1016/j.actbio.2014.09.022 PMC4278755 25242651

[96] Paredes-Juarez GA, de Haan BJ, Faas MM, de Vos P (2014). A technology platform to test the efficacy of purification of alginate. Materials.

[97] Caires HR (2016). Macrophage interactions with polylactic acid and chitosan scaffolds lead to improved recruitment of human mesenchymal stem/stromal cells: a comprehensive study with different immune cells. J. R. Soc. Interface.

[98] Bi D (2017). Alginate enhances Toll-like receptor 4-mediated phagocytosis by murine RAW264.7 macrophages. Int. J. Biol. Macromol..

[99] Xie M (2020). Immunoregulatory effects of stem cell-derived extracellular vesicles on immune cells. Front. Immunol..

[100] Lee S (2020). Mesenchymal stem cell-derived exosomes suppress proliferation of T cells by inducing cell cycle arrest through p27kip1/Cdk2 signaling. Immunol. Lett..

[101] Kerkelä E (2016). Adenosinergic immunosuppression by human mesenchymal stromal cells requires co-operation with T cells. Stem Cells.

[102] Lu L-L (2006). Isolation and characterization of human umbilical cord mesenchymal stem cells with hematopoiesis-supportive function and other potentials. Haematologica.

[103] Mennan C, Garcia J, Roberts S, Hulme C, Wright K (2019). A comprehensive characterisation of large-scale expanded human bone marrow and umbilical cord mesenchymal stem cells. Stem Cell Res. Ther..

[104] Lv FJ, Tuan RS, Cheung KM, Leung VY (2014). Concise Review: The surface markers and identity of human mesenchymal. Stem Cells Stem Cells.

[105] Mohammadi, M. R. et al. Isolation and characterization of microvesicles from mesenchymal stem cells. *Methods* **177**, 50–57 (2019). 10.1016/j.ymeth.2019.10.010 PMC7182501 31669353

[106] Rodriguez, S. et al. Characterization of chelator-mediated recovery of pancreatic islets from barium-stabilized alginate microcapsules. *Xenotransplantation* **27**, e12554 (2019). 10.1111/xen.12554 31495985

