# Supplementary material for: Exosome loaded immunomodulatory biomaterials alleviate local immune response in immunocompetent diabetic mice post islet xenotransplantation
Source: Commun Biol. 2021 Jun 3;4:685. doi: 10.1038/s42003-021-02229-4 (PMC8175379; doi:10.1038/s42003-021-02229-4)
Supplement: Supplementary file 1 — Supplementary Information [file 42003_2021_2229_MOESM1_ESM.pdf]

Supplementary Information

Exosome loaded immunomodulatory biomaterials alleviate local immune response in immunocompetent diabetic mice post islet xenotransplantation

M. Rezaa Mohammadi[1,2,3], Samuel Mathew Rodriguez[2], Jennifer Cam Luong[2,3], Shiri Li[3], Rui Cao[2,3], Hamad Alshetaiwi[4], Hien Lau[2], Hayk Davtyan[2,5], Mathew Blurton Jones[2,5,6,7], Mahtab Jafari[8], Kai Kessenbrock[4], S. Armando Villalta[7], Paul de Vos[9], Weian Zhao[2,8,10], Jonathan RT Lakey[2,3,]*

[1]Department of Materials Science and Engineering, University of California Irvine, CA, 92617, USA

[2]Sue and Bill Stem Cell Center, University of California Irvine, CA 92617, USA

[3]Department of Surgery; Department of Biomedical Engineering, University of California Irvine, CA 92868, USA

[4]Department of Biological Chemistry, University of California, Irvine, Irvine, CA 92697, USA

[5]Institute for Memory Impairments and Neurological Disorders, University of California, Irvine, Irvine, CA 92696, USA

[6]Department of Neurobiology and Behavior, University of California, Irvine, Irvine, CA 92696, USA

[7]Institute for Immunology, University of California Irvine, Irvine, CA 92697, USA

[8]Department of Pharmaceutical Sciences, University of California Irvine, Irvine, CA 92697, USA

[9]Department of Pathology and Medical Biology, section Immunoendocrinology, University of Groningen, University Medical Center Groningen, Groningen, The Netherlands

[10]Chao Family Comprehensive Cancer Center; Edwards Life Sciences Center for Advanced Cardiovascular Technology; Department of Biomedical Engineering, Department of Biological Chemistry, University of California, Irvine, Irvine, CA 92696, USA

*Correspondence to Jonathan RT Lakey (jlakey@uci.edu)

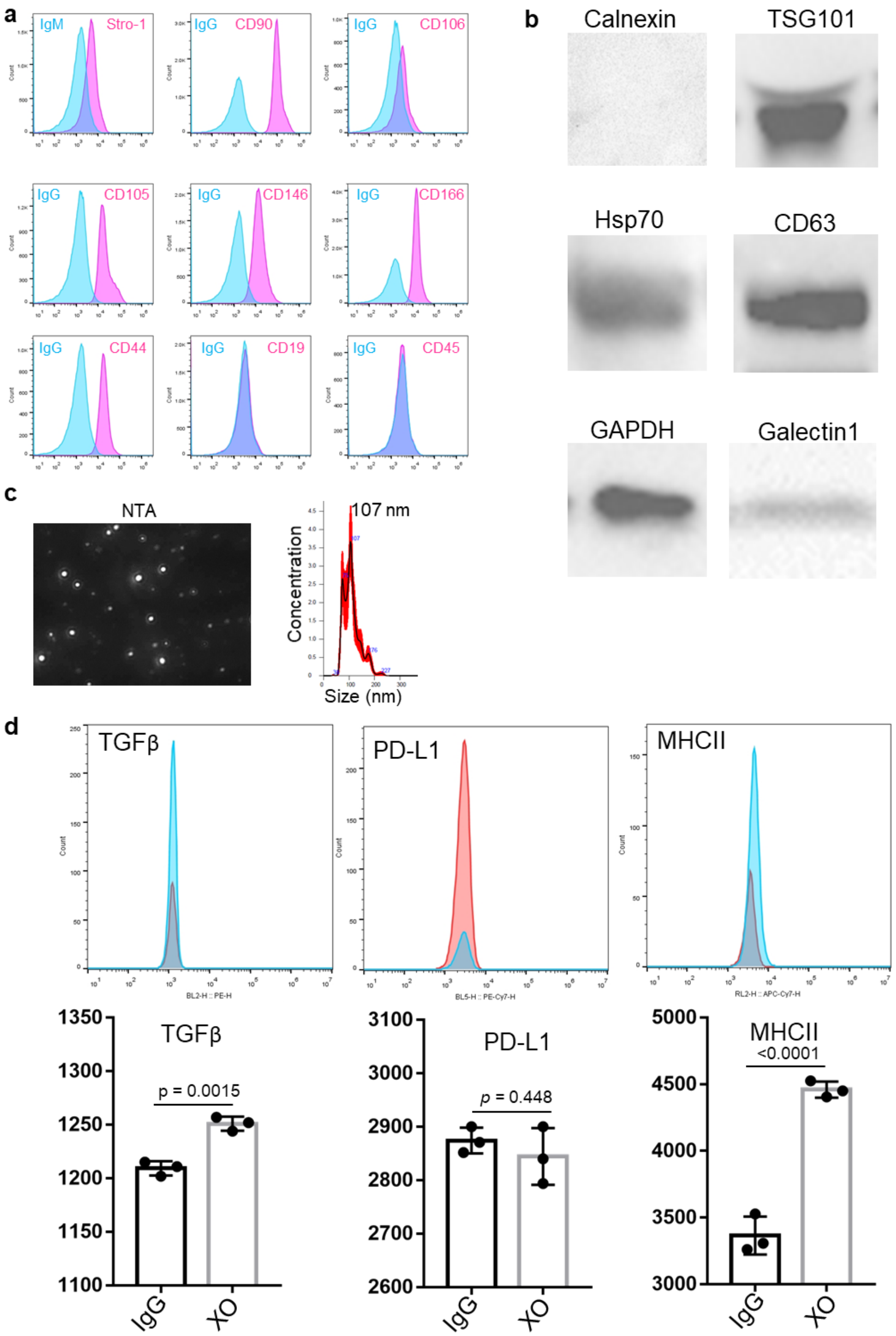

Supplementary Figure 1. Umbilical Cord Derived Stem Cells (MSCs) and their secreted XOs characterization.

a) Cells were characterized for their surface markers, showing the low expression of Stro-1, high expression CD90/Thy1, CD146/MCAM, CD105/Endoglin, CD166, CD44 while cells are negative for CD19, CD45 and CD106. Cells were then cultured as described in the Materials & Methods section, and XOs were isolated. **b)** Isolated XOs were then characterized using stablished biomarkers using Western blotting. XOs were positive for CD63, Galectin 1, TSG101, HSP70, Hsp70, and negative for and endoplasmic reticulum marker Calnexin. **c)** XOs possess spherical shape with the 105 ± 48 nm as an average size for maximum quantity of vesicles, based on NTA analyses. It should be noted that in average, $1.7 \times 10^{12} \pm 7.6 \times 10^{10}$ XOs/mL were isolated from ~ 150 to 190 million cultured MSCs in 100% confluency. **d)** Flow cytometry analysis of TGFβ, PD-L1, and MHCII expression on XOs bound to anti-CD63-coated beads. Statistical significance is calculated through unpaired t-test with Welch's correction.

We recently performed similar characterization for bone marrow derived MSC derived exosomes[1] and microvesicles[2], and found that Calnexin marker can be used as one of the markers to distinguish between exosomes and microvesicles as well as XOs purity. Comparing the Western blotting results from MSC derived MVs and MSC derived exosomes[1] suggest that calnexin and CD81 may potentially be used to distinguish between exosomes and MVs. XOs were visualized and quantified using NTA analysis, where $1.7 \times 10^{12} \pm 7.6 \times 10^{11}$ XOs/mL spherical particles with average diameter 105 ± 48 nm were isolated from ~ 150 to 190 million cultured MSCs in 100% confluency (**Supplementary Figure 1c**). In developing methods to analyze XOs, we particularly sought to measure the expression of TGFβ-1 and PD-L1, as its expression on cancer cells XOs has been suggested to play a critical role in the immune evasion of tumor microenvironment[3, 4, 5].

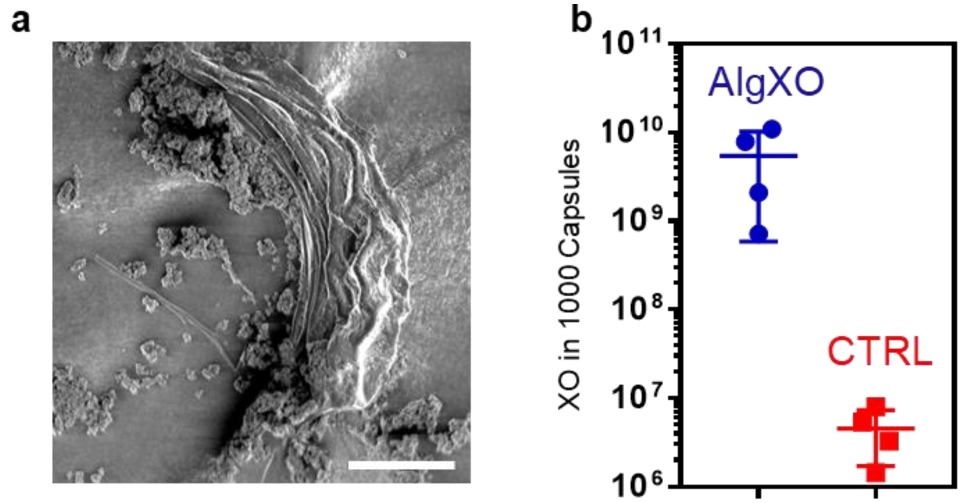

Supplementary Figure 2. a) Freeze-Fractured Scanning Electron Microscopy of an AlgXO microcapsule, showing the encapsulated XOs within the microcapsules (Scale = 100 μm). **b)** total number of exosome encapsulated within ~1000 AlgXO microcapsules is $5.43 \times 10^9 \pm 4.84 \times 10^9$ using NTA analysis. (n = 4 separate preparation)

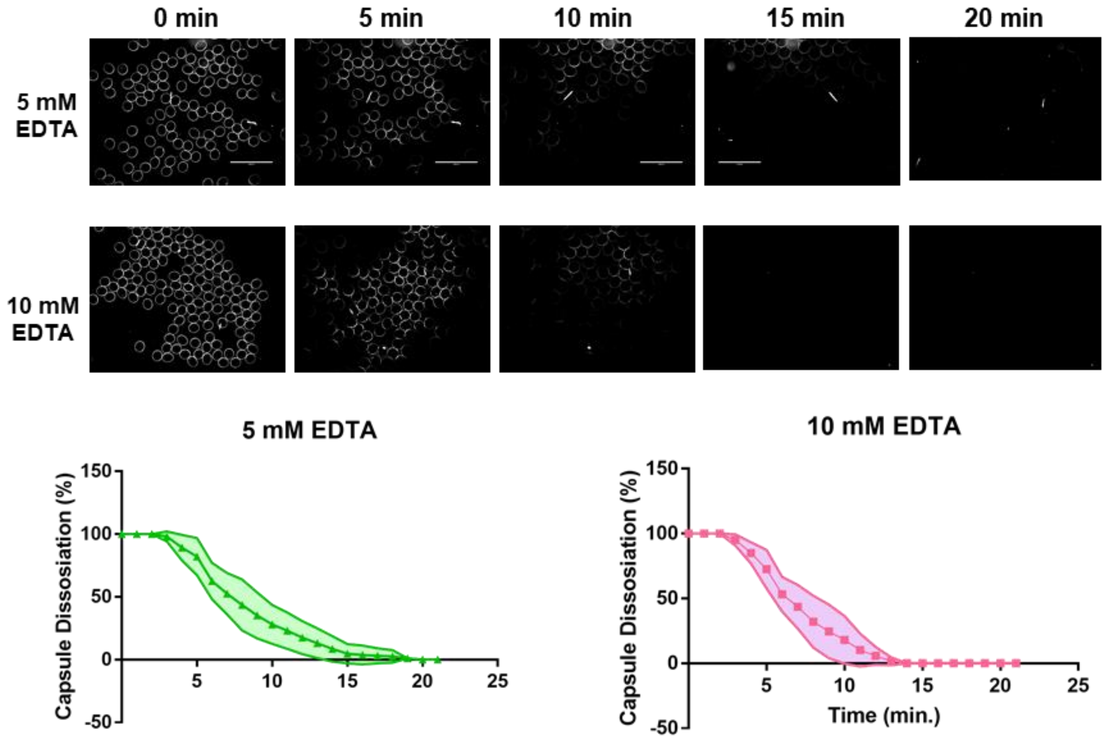

Supplementary Figure 3. EDTA dissolves alginate microcapsules. CTRL microcapsules (n = 100) were dissolved in 5 or 10 mM EDTA, and microscopic images were taken in 1 min intervals using EVOS imaging system microscope.

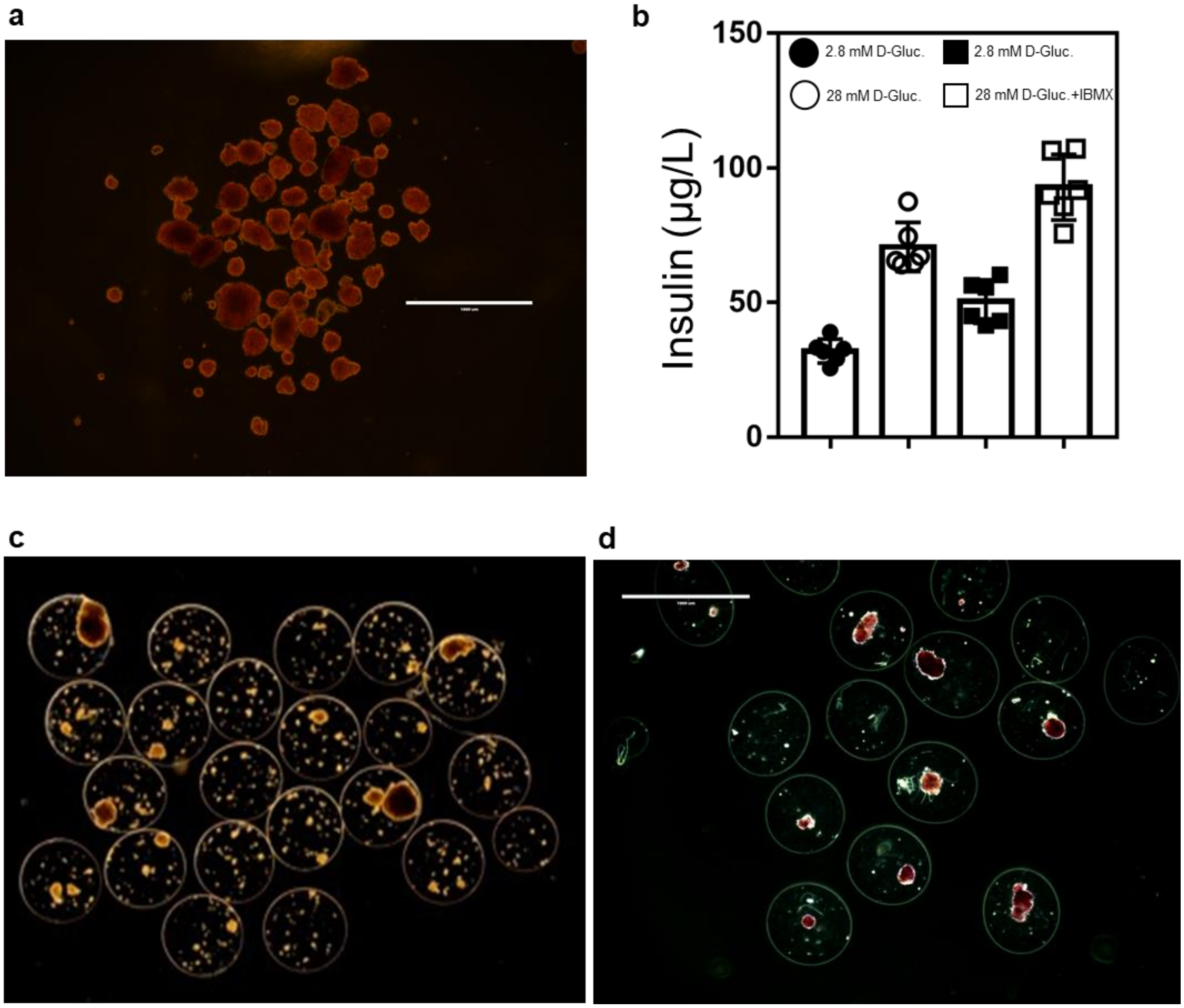

Supplementary Figure 4. Islets quality control. After each islet isolation, we ran quality control measurements. a) DTZ staining to quantify the islet purity and count (947 ± 137 IEQ). **b)** Glucose Stimulation Insulin Release (GSIR) test is run to validate the functionality of the isolated islets. Encapsulated **c)** CTRL microcapsules and **d)** AlgXO microcapsules

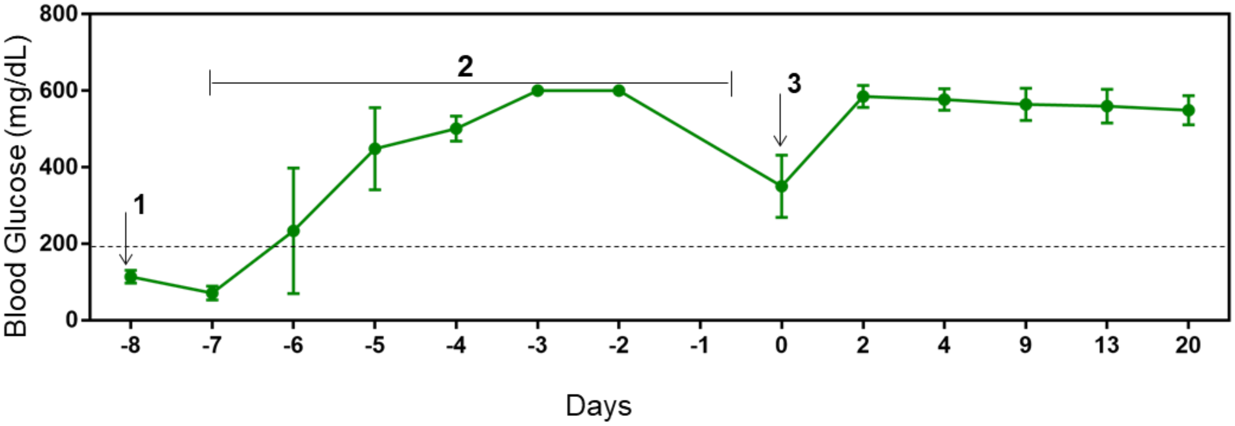

Supplementary Figure 5. Transplantation of AlgXO microcapsules without islets failed to reverse hyperglycemia in STZ mice. Empty (without islets) AlgXO microcapsules could not reverse the hyperglycemia in diabetic mice.

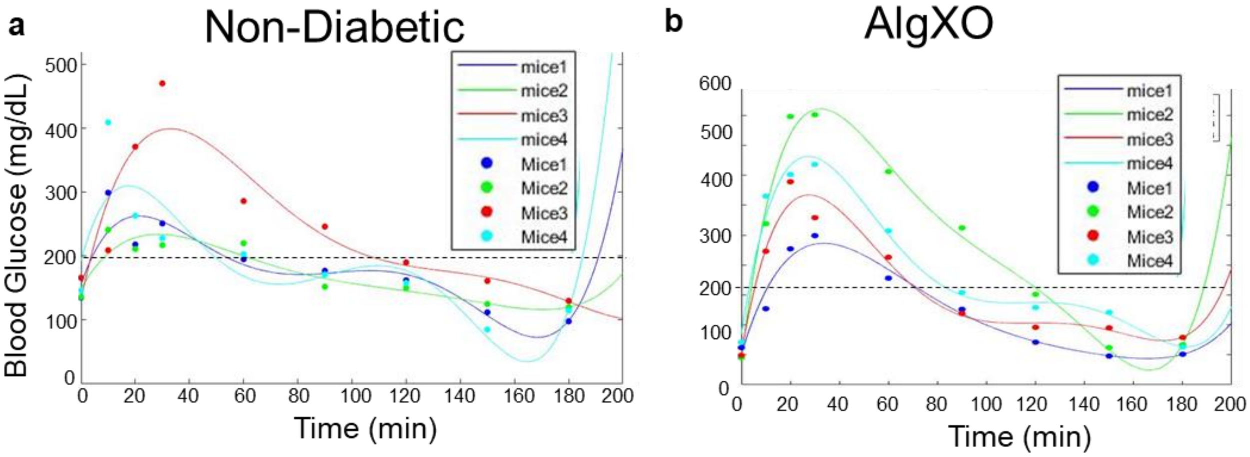

Supplementary Figure 6. Polynomial regressions onto glucose challenge response. a) Polynomials with degree 5 were assigned to the OGTT curve for 4 representative mice of a non-diabetic group and **b)** AlgXO transplanted group (1500 IEQ). Small circles show the raw OGTT data and lines represent the assigned polynomial. Dashed line demonstrates the normoglycemic criterion (i.e. blood glucose < 200 mg/mL).

Islet Dose Study

Clinical trials for islet transplantation have shown that allogeneic or xenogeneic source of islets affect the clinical efficacy, and have resulted in conflicting results. More specifically, although xenotransplantation in non-immunosuppressed diabetic patients partially reduced

hypoglycemic events, higher doses of xenogeneic islets were less effective[6, 7]. Results from this trial showed that the transplantation of 5000 IEQ/kg xeno-islets was associated with superior glycemic control and graft function compared to higher doses of xeno-islets (i.e. 15,000 or 20,000 IEQ/kg). Interestingly, a recent auto-transplantation clinical trial demonstrated a strong dose-response relationship between the islet dose and graft function[8]. This trial suggested that the islet graft failure was 25-fold more likely in patients transplanted with low dose (< 2000 IEQ/kg) islets versus higher doses (≥ 5000 IEQ/kg or more) [8]. We thus sought to understand whether such observations could be replicated in our pre-clinical diabetic mice model, and we found that the xeno-islet dose is a critical determinant of the transplant therapeutic efficacy. We used a low dose (500 IEQ) and a high dose (5000 IEQ) islets transplanted within AlgXO and CTRL microcapsules. Islets (5000 IEQ) within AlgXO reversed hyperglycemia for about 80 days but failed to do so in longer periods. Surprisingly, 5000 IEQ islets within the CTRL microcapsules were not able to consistently reverse the hyperglycemia in STZ mice (**Supplementary Figure 7a**). We further repeated the efficacy of AlgXO transplants in response to OGTT in the 5000 IEQ transplanted group and compared against non-diabetic control (**Supplementary Figure 7b**). Polynomials with degree 5 were assigned to the OGTT curve of every individual mice (**Supplementary Figure 7c**), and time to normoglycemia was calculated based on the value of 200 for the polynomial functions. **Supplementary Figure 7d** demonstrates that the average time to reach normoglycemia after an OGTT for mice with AlgXO transplants was 112 ± 32 minutes. This suggest a delay in glucose response of mice received AlgXO transplants versus non-diabetic mice ($p = 0.08$). In addition, 6 out of 10 diabetic mice that received 5000 IEQ islets within CTRL microcapsules died within a day of transplantation, while this ratio was 1 out of 8 for AlgXO group (**Supplementary Figure 7e**, $p = 0.0018$). As a result, AlgXO microcapsules delayed the graft rejection and increased the

normoglycemic duration in mice transplanted with high dose islets (**Supplementary Figure 7f**); however, with less efficacy than medium dose of islets i.e. 1500 IEQ. We further tested lower dose of islets (500 IEQ), where neither the AlgXO nor the CTRL microcapsules were able to reverse hyperglycemia in the recipient mice (**Supplementary Figure 7g**).

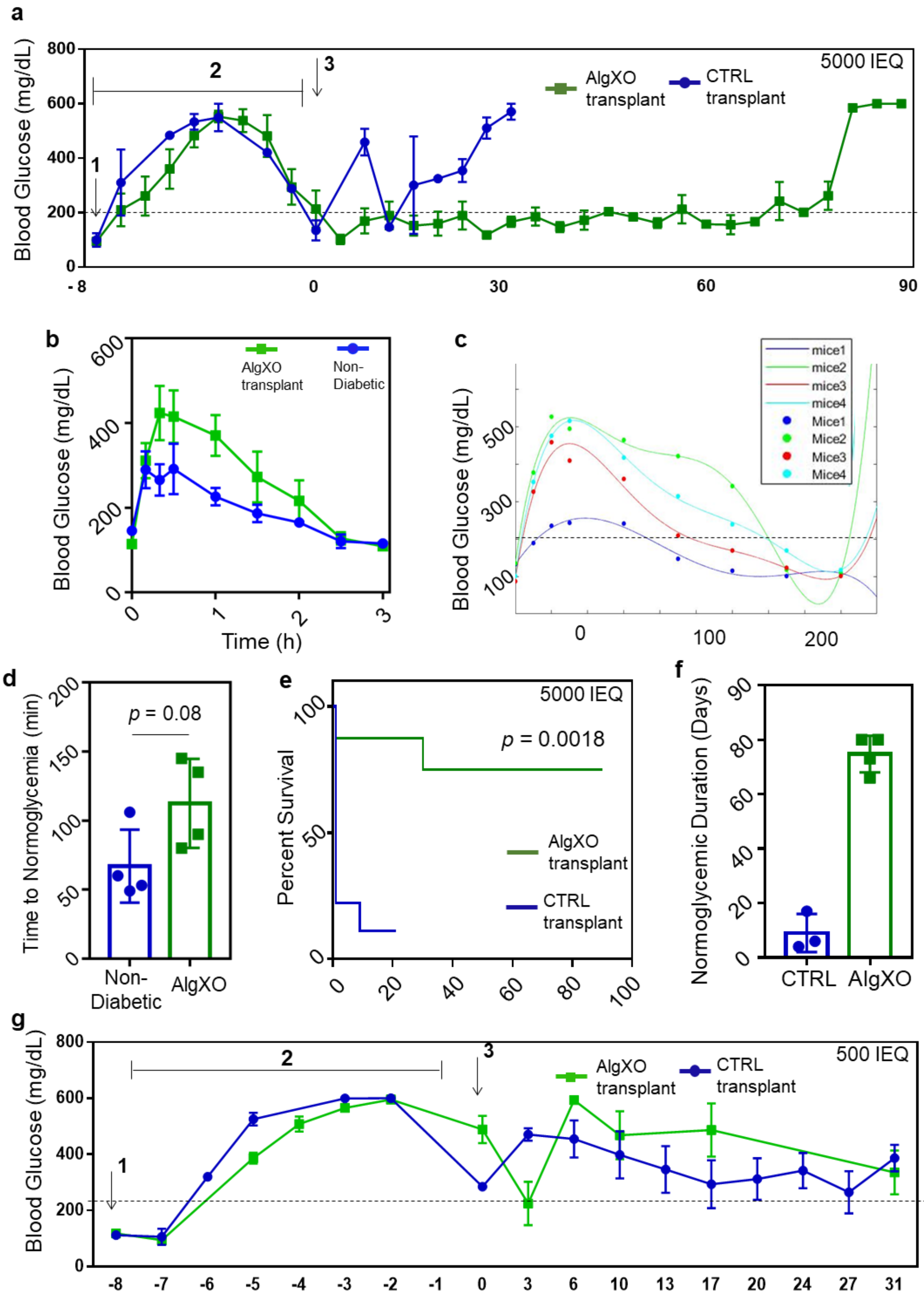

Supplementary Figure 7. Dose study of islets xenotransplantation (i.e. 500 or 5000 IEQ islets) in immunocompetent STZ mice. a) In higher islet dosage (5000 IEQ), CTRL transplants failed to consistently reverse hyperglycemia in C57/BL6 STZ mice.

However, AlgXO transplants reversed hyperglycemia for ~ 80 days. **b)** We further tested the efficacy of AlgXO transplants in response to oral glucose tolerance test (OGTT). One month after transplantation, AlgXO transplants successfully reversed hyperglycemia event induced by glucose challenge with a similar trend as non-diabetic mice. **c)** Polynomials with degree 5 were assigned to the OGTT curve of every individual mice and equations were solved to find the average time needed for the mice blood glucose to reach 200 mg/dL after an OGTT. **d)** The average time to reach normoglycemia (i.e. 200 mg/dL) after an OGTT was 112 ± 32 minutes for mice transplanted with 5000 IEQ islet encapsulated in AlgXO. The average time to reach normoglycemia for non-diabetic mice was 67 ± 26 minutes. This suggest a slight delay in glucose response of mice received AlgXO transplants versus non-diabetic mice (p = 0.08). **e)** Mice received CTRL transplants with 5000 IEQ islets had low survivals, where 6 of 10 mice died within a day of transplantation, while only 1/7 mice receiving AlgXO transplants with 5000 IEQ islets died within one day of transplantation and 5 others remained alive till the end of the study (p = 0.0018, Long-rank (Mantel-Cox) test). **f)** Mice that received AlgXO transplants with 5000 IEQ isltes remained normoglycemic for 75 ± 7 days, while this duration for CTRL transplanted mice was 9 ± 7 days after transplantation. **g)** Lower dose islets (500 IEQ) was ineffective in euglycemic induction neither within CTRL nor AlgXO microcapsules. On the diagram, 1 shows the STZ induction, 2 shows the time for diabetes progression, and 3 shows the transplantation timepoints. Statistical significance is calculated through unpaired t-test with Welch's correction.

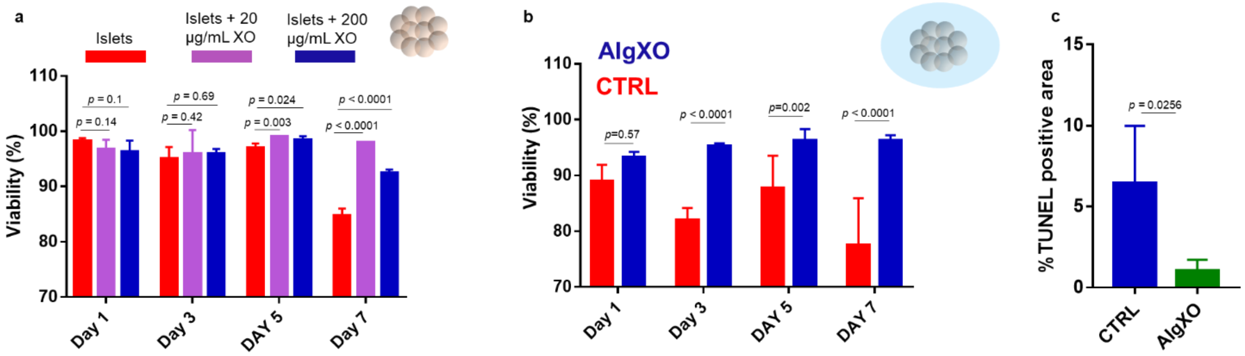

Supplementary Figure 8. XOs enhance the viability of naked and encapsulated rat islets. a) Addition of 20 µg/mL and 200 µg/mL XOs to the islet cultures significantly enhances the viability of islets after 5 and 7 days of culture. It should be noted that viability was measured using Calcein AM (live cells) and propidium iodide (dead cells) staining. **b)** Starting from 3 days of islet encapsulation, AlgXO enhances the viability of encapsulated islets within the first week of encapsulation. **c)** TUNEL assay demonstrated that after 1 month of transplantation, the TUNEL positive area of islets transplanted within AlgXO (1.02% ± 0.32%) is higher (n = 5, p = 0.0256) compared to CTRL (6.44% ± 1.59%). Statistical significance is calculated through unpaired t-test with Welch's correction.

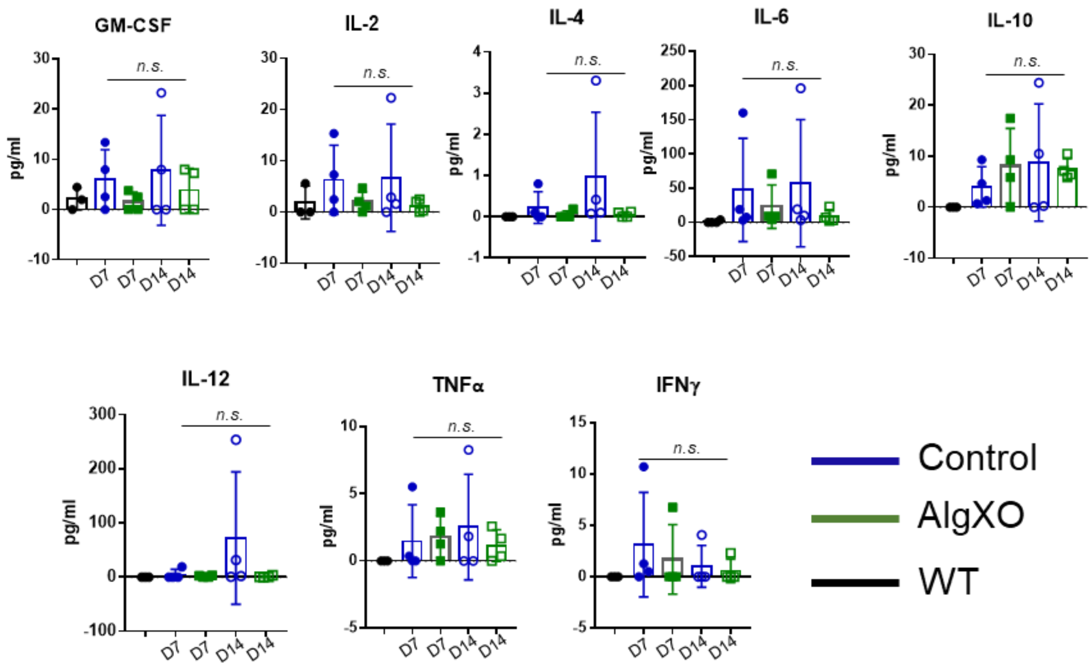

Supplementary Figure 9. Blood cytokines analyses of mice received AlgXO or CTRL microcapsules. Mice serum was harvested from mice at days 7 and 14 after transplantation, showing no significant difference among groups. One-way ANOVA was conducted to measure the statistical difference. Wiled Type (WT) mice was also added to the groups as a negative control (n = 4, statistical significance is calculated through unpaired t-test with Welch's correction).

Immune Microenvironment Around Subcutaneous Microcapsules

We found that total cell infiltration around microcapsules was significantly lower in AlgXO fibrotic tissues ($p = 0.011$). Similar trends were observed for CD68 ($p = 0.037$) and MHCII ($p = 0.015$). In contrast, there was no association between CD206 expression ($p = 0.112$). While these observations suggest the less immune-infiltrated milieu in AlgXO fibrotic microenvironment, the T cell sub population (CD3+) and fibrosis marker (αSMA) were found to be expressed more in AlgXO fibrotic microenvironment. These mixed outcomes were against our initial hypothesis on the anti-inflammatory and/or anti-fibrotic response of AlgXO microcapsules *in vivo*. In particular, αSMA, which was highly expressed in the AlgXO microenvironment, is a marker for activated myofibroblasts that are responsible for downstream collagen deposition and fibrosis of implanted alginate microcapsules [9]. However, αSMA is also a contractile protein

expressed in pericytes as well as in the vascular smooth muscle cells that surround arteries and arterioles [10]. In the histological observations, the αSMA cells were found to have a round structure consistent with blood vessel structure (**Figure 2e**). Next, we quantified the blood vessel formation and found that there is more blood vessel within the subcutaneous area (and around microcapsules) of AlgXO 2-weeks explants (**Supplementary Figure 10a**). We further isolated cells from fibrotic tissues and analyzed their subpopulation using flow cytometry. There was significantly higher CD45+ cells (n = 4; $p < 0.0001$) collected from AlgXO fibrotic tissues (33.1% ± 8.0%) compared to control (83.0% ± 12.8%) (**Supplementary Figure 10b**). Tissue sections were further analyzed for αSMA showing vasculature presence in the AlgXO fibrotic microenvironment, demonstrating a vascular-shaped microstructure (**Supplementary Figure 10c, d**). These results in their totality suggest the presence of blood vasculature and less inflammatory milieu around AlgXO fibrotic tissues.

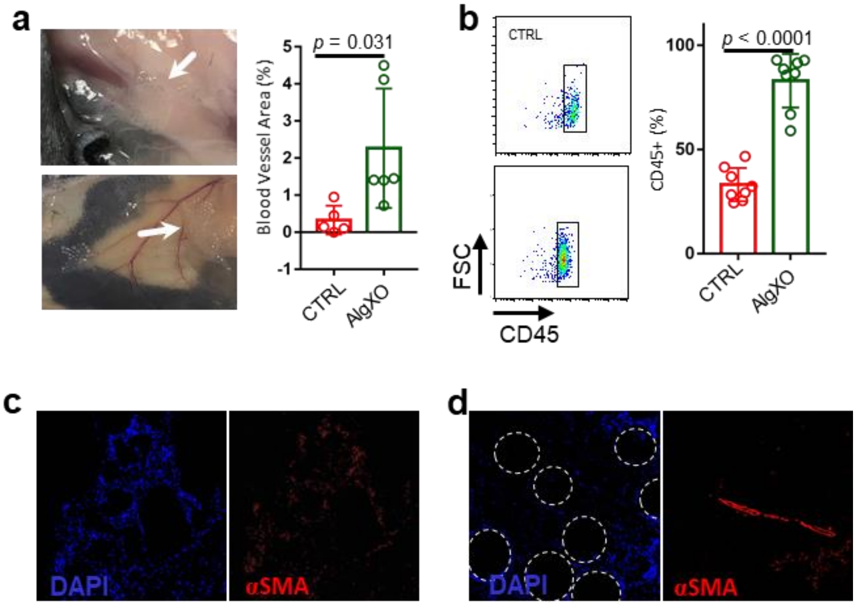

Supplementary Figure 10. Vasculature present in the fibrotic tissue around AlgXO. a) Pictures from subcutaneous explants show presence of blood vessels in AlgXO fibrotic microenvironment. **b)** Flow cytometry analyses shows the presence of higher

CD45+ cells (p < 0.0001) harvested from AlgXO fibrotic tissues (33.1% ± 8.0%) compared to control (83.0% ± 12.8%). **c)** αSMA (markers of blood vessels) were absent in CTRL fibrotic tissues compared to **d)** AlgXO. Statistical significance is calculated through unpaired t-test with Welch's correction.

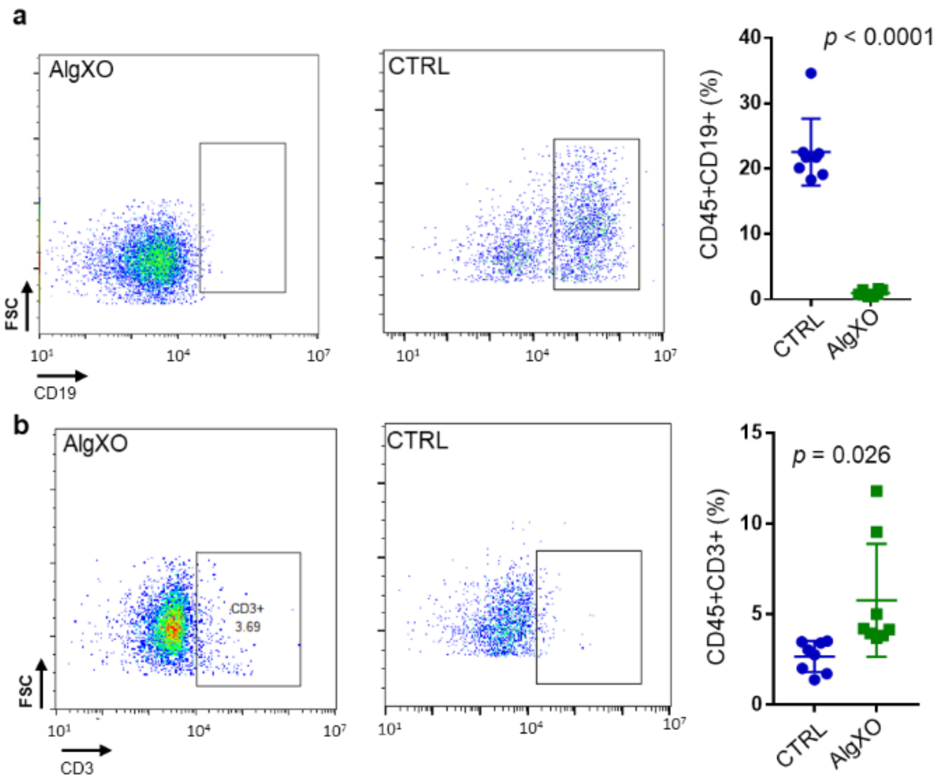

Supplementary Figure 11. The percentage of **a)** B and **b)** T cells presence in the fibrotic Tissues around AlgXO and CTRL. (n = 4). Statistical significance is calculated through unpaired t-test with Welch's correction.

We pursued the experiments to further investigate the anti-inflammatory properties of AlgXO microcapsules. Immune infiltration could be characterized with cell types present in the lavage around the inflamed area, particularly for biomaterials-based inflammation [11, 12]. Live cells within the subcutaneous lavage were first analyzed for common lymphocyte marker (CD45). **Supplementary Figure 12a, b** shows the percentage of CD45+ cells in lavage of CTRL implanted mice were 41.6% ± 4.2% and in the AlgXO implanted were 8.5% ± 5.2% (n = 4, $p < 0.0001$). Sub-gating on CD45+ cells, the percentage of CD11b+ cells decreased from 68.4% ± 9.4% for CTRL

to 17.6% ± 13.4% for AlgXO microcapsules ($p < 0.0001$). Around 68.2% ± 9.5% of CD11b+ cells are also expressing MHCII for CTRL, while this percentage is 23.5% ± 16.3% for AlgXO microcapsules ($p < 0.0001$). Interestingly, there was no detectable CD45+CD11b+MHCII-CD206+ (M2-like macrophages [13]) for CTRL, while these macrophages 3.7% ± 1.9% for lavage retrieved from the surrounding environment of AlgXO microcapsules ($p < 0.0001$).

To gain a more holistic information on the lavage immune-profile, we compared the lavage components of both microcapsules at the cellular level through tSNE representation. **Supplementary Figure 12c, d** show tSNE plots and two sub-populations that were analyzed for immune markers. Query 1 (gated on specific sub population present in CTRL but not in AlgXO) was CD45+CD11b+CD3-CD19-MHCII-Ly6C-Ly6G-, which is likely to be non-activated dendritic cells [14]. Query 2 (gated on specific sub population present in AlgXO but not in CTRL) showed the subpopulation of cells with CD45-CD11b-CD3-CD19-MHCII-Ly6C-Ly6G- markers, which are likely to be from neither myeloid nor lymphoid origin. These results in their totality supports the reduced-inflammatory response against AlgXO implants, while non-inflammatory tissues were formed around AlgXO. It should be noted that transplantation of 1500 IEQ rat islets within AlgXO or CTRL failed to regulate the dysglycemia when transplanted subcutaneously (**Supplementary Figure 13**). Interestingly, while 1500 IEQ islets regulated mice hyperglycemia when transplanted intraperitoneally, subcutaneous transplantation failed to do so. A combination of stronger fibrotic response, lack of moveability, and more hypoxic environment in the subcutaneous area are likely among the reasons for such a difference.

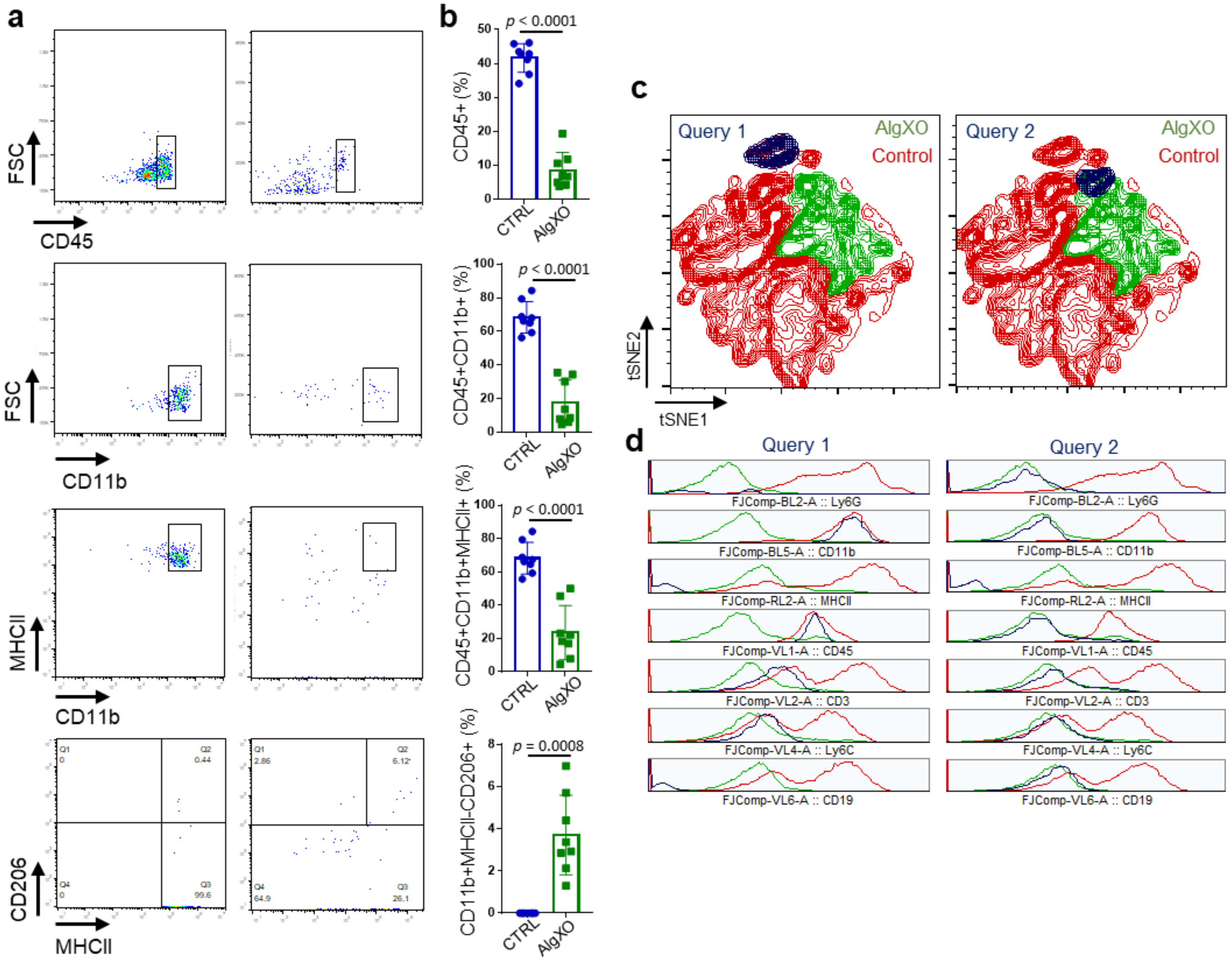

Supplementary Figure 12. Flow cytometry analyses of lavage around microcapsules show distinct immunocytes population around AlgXO and CTRL microcapsules. a, b) Flow cytometry analyses demonstrates the total CD45+ population present around AlgXO is less than CTRL microcapsules. Similar trend was observed for CD11b+, CD11b+MHCII+, and CD11b+MHCII-CD206+ sub-populations. **c)** tSNE plots demonstrates the different cell environment present in the lavage collected from surrounding non/low adherent cells around AlgXO and CTRL explants. **d)** Two sub-populations were then analyzed for immune markers. Cells in Query 1 (gated on specific sub population present in CTRL but not in AlgXO) was CD45+CD11b+CD3-CD19-MHCII-Ly6C-Ly6G-, which is likely to be dendritic cells. Cells in Query 2 (gated on specific sub population present in AlgXO but not in CTRL) was CD45-CD11b-CD3-CD19-MHCII-Ly6C-Ly6G-, which is likely to be neither from myeloid or lymphoid origin. (n = 4, statistical significance is calculated through unpaired t-test with Welch’s correction)

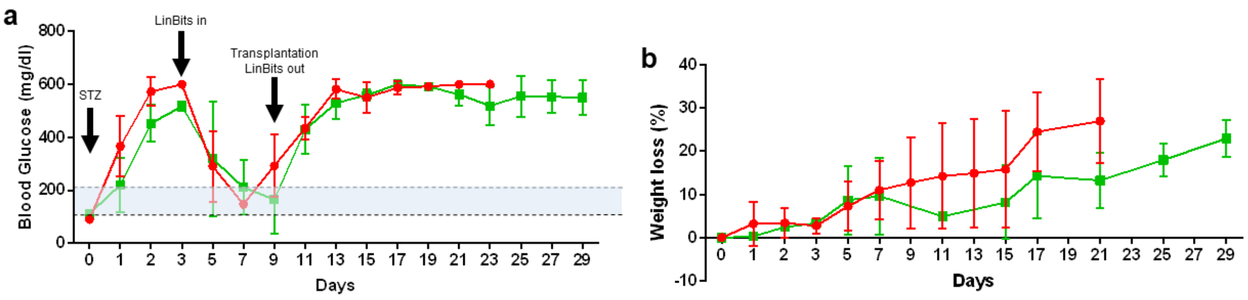

Supplementary Figure 13. Subcutaneous transplantation of islets encapsulated in either CTRL and AlgXO. The **a)** glucose and **b)** body weights of STZed mice were tracked for a month, and there was no significant improvement in the glycemic control in any of the groups.

Simulation of release model for nanoparticle

To better understand the spatiotemporal profiles for the controlled release of XOs from AlgXO, we simulated such release using a MATLAB code. Simulations were run for homogenous spatially distributed XOs within AlgXO with diameter of 300 µm. Due to the 50-150 nm size distribution of XOs, we run the simulation with 50, 100, and 150 nm nanoparticle size. To further validate and characterize the release profiles other sizes (i.e. 10, 200, and 500 nm) were also tested in our simulation model. The 2.5% (weight/volume) alginate was used in our experimental studies, thus, we used the same percentage for the porosity of alginate calculations (equation 1):

<u>Modeling assumptions</u>

1. *Microcapsule size and porosity*

Simulations were run for uniformly sized and spatially distributed microcapsules and diameter was assumed to be 300 µm. Value of 2% (weight /volume) is evaluated as the default value for all simulations. Porosity of alginate solid is calculated by equation 1.

$$\text{porosity} = \frac{\rho 1-\rho 2}{\rho 1-\rho 3} \qquad (1)$$

ρ1 is particle density, $\rho 2$ is bulk density, and ρ3 is fluid density. For alginate solid, particle density is 1.6 g/ml, fluid density is density of water, which is 1g/ml. Bulk density will be based on the concentration of alginate, which is shown in equation 2.

$$\rho 2 = \frac{100 + C_{alg}}{100} \qquad (2)$$

2. Surrounding media

Assuming that capsules are implanted and surrounded by physiological fluid, surrounding viscosity was chosen to be $3.5*10^{-3}$ Pa*s.

3. Temperature

Assuming the capsule are implanted, temperature of microcapsule and surrounding environment should be close to body temperature, which is 37 degree.

4. XOs concentration

XOs are homogeneously mixed inside the microcapsules, with an initial concentration of 10 particles/μm^3.

5. XOs size

XOs are generally recognized to be between 30-150 nm. Thus, we set up the particle diameter as 10 nm, 50 nm, 100 nm, 200 nm, and 500 nm to see the different result as particle size changes. Particles larger than 450 nm cannot diffuse out from the capsule [18].

6. Diffusion model

To simplify our modeling, we just assumed that XOs diffuse out of microcapsules based on gradient density differences. We also assumed that a uniformed sphere symmetrically

diffuses out in any direction. Thus, we established a 1-dimensional diffusion model for XOs.

Mathematical model assumptions

1. *1-D diffusion equation*

We are using heat equation to calculate the 1-dimensional diffusion of nanoparticles. Heat equation is a partial differential equation as shown in equation 3.

$$\frac{\partial \mathrm{C}}{\partial \mathrm{t}} = D * \frac{\partial^2 C}{\partial x^2} \qquad (3)$$

C is concentration gradient, t is time, and x is distance from center of the capsule. D is diffusion coefficient of XOs at certain position.

2. *Diffusion coefficient outside the microcapsule*

To determine diffusion coefficient outside the capsule, we use Stroke-Einstein equation (equation 4).

$$\mathrm{D} = \frac{\mathrm{R}}{\mathrm{N}_A} * \frac{\mathrm{T}}{6*\pi*\eta*r} \qquad (4)$$

Where R is gas constant, NA is Avogadro constant, T is temperature in kelvin, η is viscosity of solution, and r is radius of XOs.

3. *Effective diffusion coefficient inside the capsule*

Effective diffusion coefficient inside a porous media is largely based on porosity and tortuosity of media. Generally, effective diffusion coefficient can be calculated based on equation 5.

$$D_{eff} = D * \frac{porosity}{tortuosity} \qquad (5)$$

For porous media, normally we have a relationship between porosity and tortuosity, which is shown in equation 6.

$$tortuosity = porosity^{-\frac{1}{3}} \quad (6)$$

Since the microcapsule diameter is ~150 μm, the program will simulate concentration gradient from 0 to 500 μm. A 600 s run time was selected to visualize the concentration change inside the area. Diffusion of nanoparticle for 10 nm, 50 nm, 100 nm, 200 nm, and 500 nm is plotted (**Figure 3h**). As shown in the graph, nanoparticles with smaller diameter diffuse faster than which with larger diameter (**Supplementary Figure 14**). Particle with 500 nm diameter cannot diffuse out of the capsule. For particles with size of 10 nm, within 600 s, concentration of particles at center of the capsule will drop to 40% of initial concentration. For 50 nm nanoparticles, concentration at center of the capsule will drop to 80% of initial value. For 100 and 200 nm, there is no significant drop of concentration at center of the microcapsule. Concentration of nanoparticle outside the capsule also depends on particle size. For 10 nm nanoparticle, after 600 s, concentration of nanoparticle outside the capsule will larger than 15 particles/μm^3. For 50nm, 100nm, and 200nm, there is not enough nanoparticles 350 μm from center of the capsule (concentration < 1 particle/μm^3).

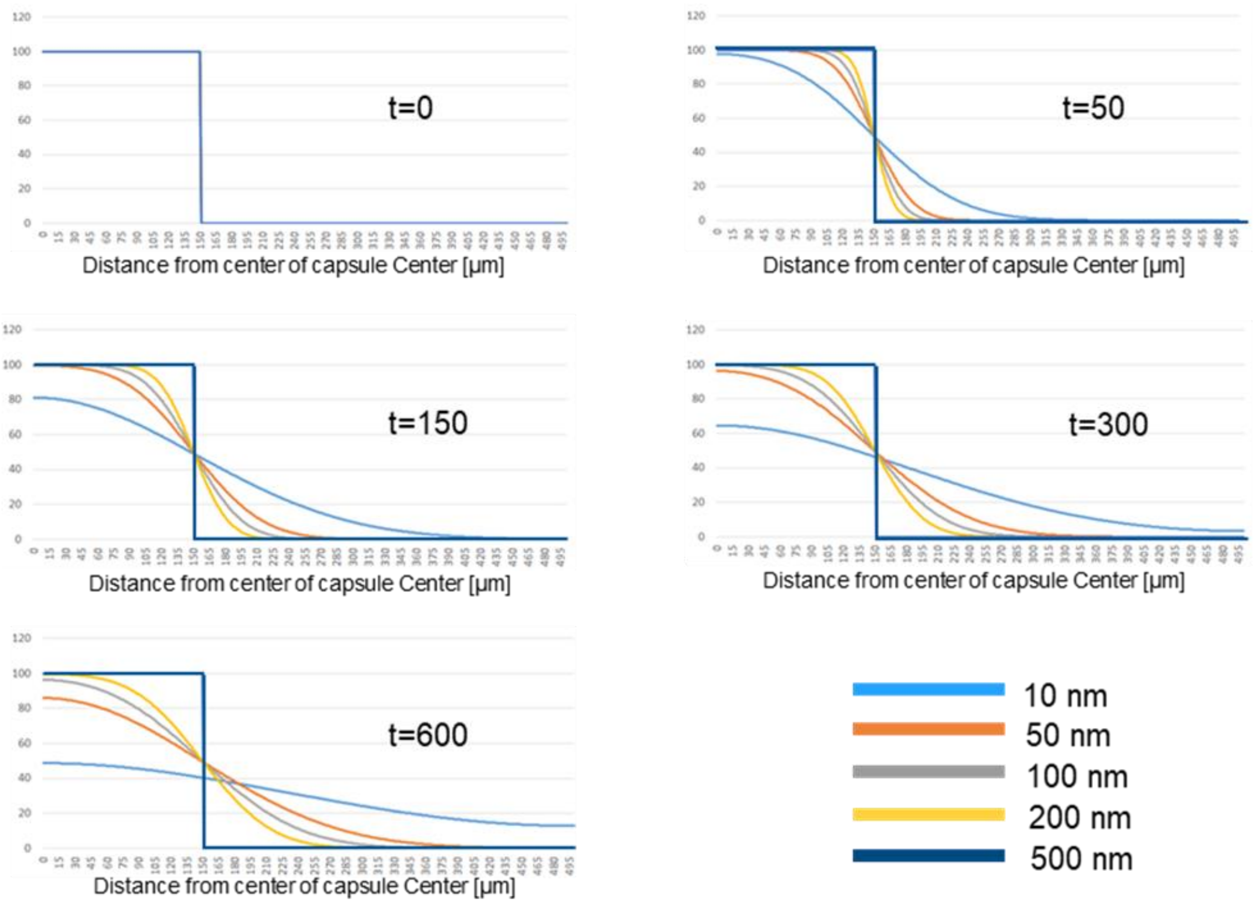

Supplementary Figure 14. Simulated controlled release of particles with 10, 50, 100, 200, or 500 nm of diameters. At t > 0, particles with diameter ≤ 200 nm show diffusion profiles, where smaller particles diffuse faster. Particles with diameter of 500 nm do not show diffusion out of microcapsules at least for 600 s.

Through our high-throughput cytokine assay, we found that XOs significantly reduce the production of G-CSF, IFNγ, LIF, KC, MIP-2, RANTES, IL-6, LIX, and VEGEF from LPS stimulated macrophages (**Figure 4e**). These cytokines and chemokines are hallmarks of NFκB inflammatory pathway, suggesting that XOs likely possess anti-inflammatory properties through regulating this pathway. These cytokines could have complementary effects. For example, chemotactic signals include CXC chemokines such as CXCL1/KC, CXCL2/MIP-2, and CXCL5/LIX, and CXCL8/IL-8, which are potent chemoattractant for NGs and their increased

production causes neutrophil granulocytes infiltration and extravasation [15]. **Supplementary Table 1** summarizes the function of these cytokines and their relation to NFκB pathway.

Supplementary Table 1. Macrophages Cytokines Influenced by XOs

**Chemokine/ Cytokine**	**Function**	**Ref.**
G-CSF/ CSF3	• Regulates the survival, maturation, and proliferation of neutrophil progenitors<br>• Regulates the differentiation of granulocyte lineages<br>• Regulates neutrophils mobilization from bone marrow to peripheral tissues<br>• LPS-activated ERK2 functions by remodeling local chromatin, interacting with C/EBPβ and synergizing its transactivation activity to increase G-CSF expression	16, 17
IFNγ	• The only known type II interferon<br>• Upon binding to receptor, JAK1 and JAK2 are activated and phosphorylate STAT1<br>• Macrophages secrete upon stimulation with LPS	18, 19
LIF	• LIF acts in an autocrine manner via LIF receptor to promote STAT4 activation. Activated STAT4 together with NF-kB/p65-p52 and C/EBPb enhances IL-6 transcription	20
MIP-2/ CXCL2	• Important chemokine for recruitment of neutrophils<br>• NF-κB activation is required for MIP-2 gene expression in the LPS-signaling pathway A MIP-2 promoter could be activated by ectopical expression of NF-κB p65 or c-Jun transcription factors.	21
RANTES	• Secretes via LPS-induced NF-kB activation in monocytes through sterile α and HEAT/Armadillo motif-containing protein (SARM)toll/IL-1R domain–containing adaptor. SARM is critical for the recruitment of transcription factors and of RNA polymerase II to the Ccl5 promoter	22, 23
LIX/ CXCL5	• Important chemokine in Neutrophil trafficking	24, 25, 26

KC/ CXCL1	• CXCL1 is regulated through interactions of NF-κB with other transcriptional regulatory molecules such as poly(ADP-ribose) polymerase-1 (PARP-1) and cAMP response element binding protein (CREB)-binding protein	27, 28
VEGF	• Important protein for angiogenesis<br>• VEGF production in human macrophages is NF-κB dependent and could be significantly reduced using the NF-κB inhibitor, IκBα	29

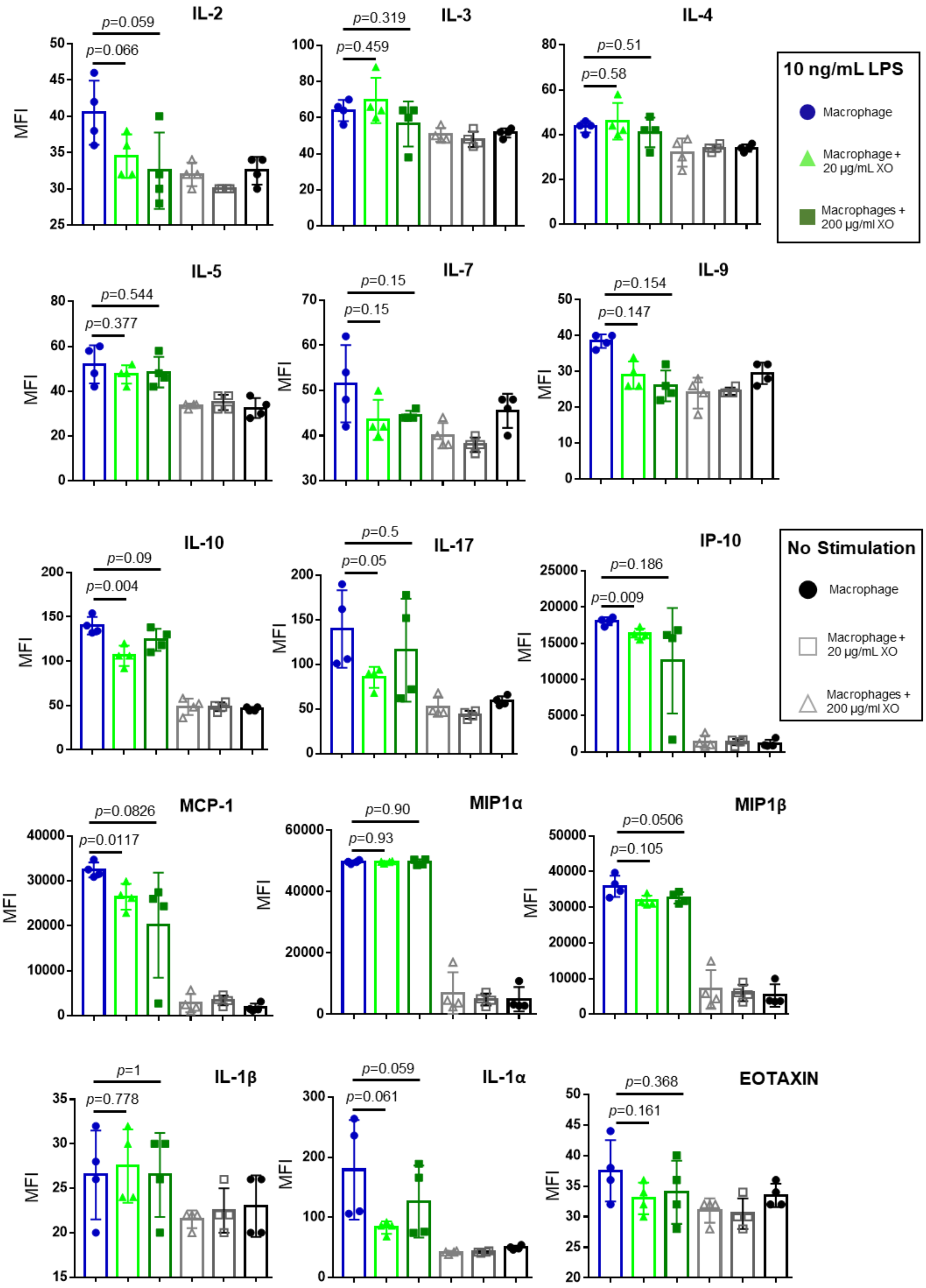

Supplementary Figure 15. XOs effect on the production of cytokines from 10 ng/mL LPS (TLR4 agonist) stimulated macrophages. Statistical significance is calculated through unpaired t-test with Welch's correction (n = 4).

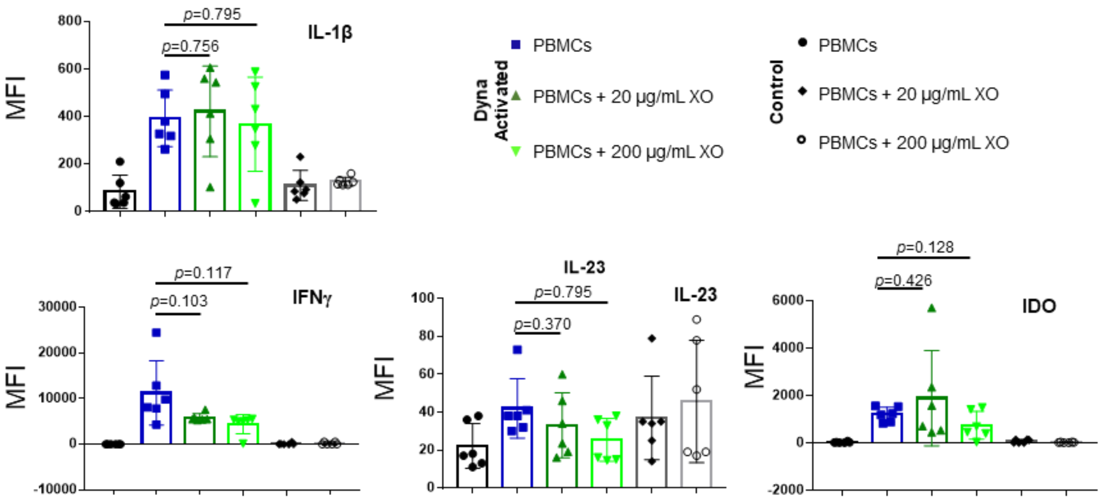

Supplementary Figure 16. Cytokines analyses from co-cultures of human activated PBMCs. PBMCs were activated with bead-bound CD3/CD28 antibodies in the presence and absence of XOs. XOs in both 20 and 200 µg/mL concentrations slightly influenced the production of IL-1β, IL-23, IFNγ, and IDO (n = 4). Statistical significance is calculated through unpaired t-test with Welch's correction.

References

1. Riazifar M, *et al.* Stem Cell-Derived Exosomes as Nanotherapeutics for Autoimmune and Neurodegenerative Disorders. *ACS Nano* **13**, 6670-6688 (2019).

2. Mohammadi MR, *et al.* Isolation and characterization of microvesicles from mesenchymal stem cells. *Methods*, (2019).

3. Daassi D, Mahoney KM, Freeman GJ. The importance of exosomal PDL1 in tumour immune evasion. *Nature Reviews Immunology*, (2020).

4. Chen G, *et al.* Exosomal PD-L1 contributes to immunosuppression and is associated with anti-PD-1 response. *Nature* **560**, 382-386 (2018).

5. Haderk F, *et al.* Tumor-derived exosomes modulate PD-L1 expression in monocytes. *Science Immunology* **2**, eaah5509 (2017).

6. Matsumoto S, *et al.* Clinical Porcine Islet Xenotransplantation Under Comprehensive Regulation. *Transplantation Proceedings* **46**, 1992-1995 (2014).

7. Ekser B, Bottino R, Cooper DKC. Clinical Islet Xenotransplantation: A Step Forward. *EBioMedicine* **12**, 22-23 (2016).

8. Chinnakotla S, *et al.* Factors Predicting Outcomes After a Total Pancreatectomy and Islet Autotransplantation Lessons Learned From Over 500 Cases. *Annals of Surgery* **262**, 610–622 (2015).

9. Doloff JC, *et al.* Colony stimulating factor-1 receptor is a central component of the foreign body response to biomaterial implants in rodents and non-human primates. *Nature Materials* **16**, 671 (2017).

10. Kornfield TE, Newman EA. Regulation of Blood Flow in the Retinal Trilaminar Vascular Network. *The Journal of Neuroscience* **34**, 11504 (2014).

11. Vegas AJ, *et al.* Combinatorial hydrogel library enables identification of materials that mitigate the foreign body response in primates. *Nature Biotechnology* **34**, 345 (2016).

12. Vegas AJ, *et al.* Long-term glycemic control using polymer-encapsulated human stem cell–derived beta cells in immune-competent mice. *Nature Medicine* **22**, 306-311 (2016).

13. Vlahos AE, Cober N, Sefton MV. Modular tissue engineering for the vascularization of subcutaneously transplanted pancreatic islets. *Proceedings of the National Academy of Sciences* **114**, 9337–9342 (2017).

14. Hey Y-Y, Tan JKH, O'Neill HC. Redefining Myeloid Cell Subsets in Murine Spleen. *Front Immunol* **6**, 652 (2016).

15. Amanzada A, Moriconi F, Mansuroglu T, Cameron S, Ramadori G, A Malik I. Induction of chemokines and cytokines before neutrophils and macrophage recruitment in different regions of rat liver after TAA administration. *Laboratory Investigation* **94**, 235-247 (2014).

16. Lieschke GJ, *et al.* Mice lacking granulocyte colony-stimulating factor have chronic neutropenia, granulocyte and macrophage progenitor cell deficiency, and impaired neutrophil mobilization. *Blood* **84**, 1737-1746 (1994).

17. Chang S-F, Lin S-S, Yang H-C, Chou Y-Y, Gao J-I, Lu S-C. LPS-Induced G-CSF Expression in Macrophages Is Mediated by ERK2, but Not ERK1. *PLOS ONE* **10**, e0129685 (2015).

18. Fultz MJ, Barber SA, Dieffenbach CW, Vogel SN. Induction of IFN-γ in macrophages by lipopolysaccharide. *International Immunology* **5**, 1383-1392 (1993).

19. Platanias LC. Mechanisms of type-I- and type-II-interferon-mediated signalling. *Nature Reviews Immunology* **5**, 375-386 (2005).

20. Nguyen HN, *et al.* Autocrine Loop Involving IL-6 Family Member LIF, LIF Receptor, and STAT4 Drives Sustained Fibroblast Production of Inflammatory Mediators. *Immunity* **46**, 220-232 (2017).

21. Kim D-S, Ho Han J, Kwon H-J. NF-κB and c-Jun-dependent regulation of macrophage inflammatory protein-2 gene expression in response to lipopolysaccharide in RAW 264.7 cells. *Molecular Immunology* **40**, 633-643 (2003).

22. Karlsen A, *et al.* Anthocyanins Inhibit Nuclear Factor-κB Activation in Monocytes and Reduce Plasma Concentrations of Pro-Inflammatory Mediators in Healthy Adults. *The Journal of Nutrition* **137**, 1951-1954 (2007).

23. Gürtler C, *et al.* SARM Regulates CCL5 Production in Macrophages by Promoting the Recruitment of Transcription Factors and RNA Polymerase II to the Ccl5 Promoter. *The Journal of Immunology* **192**, 4821 (2014).

24. Lin M, Carlson E, Diaconu E, Pearlman E. CXCL1/KC and CXCL5/LIX are selectively produced by corneal fibroblasts and mediate neutrophil infiltration to the corneal stroma in LPS keratitis. *Journal of leukocyte biology* **81**, 786-792 (2007).

25. Wang L-Y, Tu Y-F, Lin Y-C, Huang C-C. CXCL5 signaling is a shared pathway of neuroinflammation and blood-brain barrier injury contributing to white matter injury in the immature brain. *J Neuroinflammation* **13**, 6-6 (2016).

26. Chandrasekar B, *et al.* Chemokine-Cytokine Cross-talk THE ELR+ CXC CHEMOKINE LIX (CXCL5) AMPLIFIES A PROINFLAMMATORY CYTOKINE RESPONSE VIA A PHOSPHATIDYLINOSITOL 3-KINASE-NF-κB PATHWAY. *Journal of Biological Chemistry* **278**, 4675-4686 (2003).

27. Bhattacharyya S, Borthakur A, Dudeja PK, Tobacman JK. Lipopolysaccharide-induced activation of NF-κB non-canonical pathway requires BCL10 serine 138 and NIK phosphorylations. *Exp Cell Res* **316**, 3317-3327 (2010).

28. Amiri KI, Richmond A. Fine tuning the transcriptional regulation of the CXCL1 chemokine. *Prog Nucleic Acid Res Mol Biol* **74**, 1-36 (2003).

29. Kiriakidis S, Andreakos E, Monaco C, Foxwell B, Feldmann M, Paleolog E. VEGF expression in human macrophages is NF-κB-dependent: studies using adenoviruses expressing the endogenous NF-κB inhibitor IκBα and a kinase-defective form of the IκB kinase 2. *Journal of Cell Science* **116**, 665 (2003).
