# Supplementary material for: Exosome loaded immunomodulatory biomaterials alleviate local immune response in immunocompetent diabetic mice post islet xenotransplantation
Source: Commun Biol. 2021 Jun 3;4:685. doi: 10.1038/s42003-021-02229-4 (PMC8175379; doi:10.1038/s42003-021-02229-4)
Supplement: Supplementary file 2 — Description of Supplementary Files [file 42003_2021_2229_MOESM2_ESM.pdf]

Description of Additional Supplementary Files

File name: Supplementary Data 1

Description: Raw data for the main figures.
