# Supplementary material for: Exosome loaded immunomodulatory biomaterials alleviate local immune response in immunocompetent diabetic mice post islet xenotransplantation
Source: Commun Biol. 2021 Jun 3;4:685. doi: 10.1038/s42003-021-02229-4 (PMC8175379; doi:10.1038/s42003-021-02229-4)
Supplement: Supplementary file 4 — Reporting Summary [file 42003_2021_2229_MOESM4_ESM.pdf]

nature research

Corresponding author(s): Jon Lakey

Last updated by author(s): May 2, 2021

# Reporting Summary

Nature Research wishes to improve the reproducibility of the work that we publish. This form provides structure for consistency and transparency in reporting. For further information on Nature Research policies, see our Editorial Policies and the Editorial Policy Checklist.

## Statistics

For all statistical analyses, confirm that the following items are present in the figure legend, table legend, main text, or Methods section.

| n/a | Confirmed | |
|---|---|---|
| ☐ | ☒ | The exact sample size (*n*) for each experimental group/condition, given as a discrete number and unit of measurement |
| ☐ | ☒ | A statement on whether measurements were taken from distinct samples or whether the same sample was measured repeatedly |
| ☐ | ☒ | The statistical test(s) used AND whether they are one- or two-sided<br>*Only common tests should be described solely by name; describe more complex techniques in the Methods section.* |
| ☒ | ☐ | A description of all covariates tested |
| ☐ | ☒ | A description of any assumptions or corrections, such as tests of normality and adjustment for multiple comparisons |
| ☒ | ☐ | A full description of the statistical parameters including central tendency (e.g. means) or other basic estimates (e.g. regression coefficient) AND variation (e.g. standard deviation) or associated estimates of uncertainty (e.g. confidence intervals) |
| ☐ | ☒ | For null hypothesis testing, the test statistic (e.g. *F*, *t*, *r*) with confidence intervals, effect sizes, degrees of freedom and *P* value noted<br>*Give P values as exact values whenever suitable.* |
| ☒ | ☐ | For Bayesian analysis, information on the choice of priors and Markov chain Monte Carlo settings |
| ☒ | ☐ | For hierarchical and complex designs, identification of the appropriate level for tests and full reporting of outcomes |
| ☒ | ☐ | Estimates of effect sizes (e.g. Cohen's *d*, Pearson's *r*), indicating how they were calculated |

*Our web collection on statistics for biologists contains articles on many of the points above.*

## Software and code

Policy information about availability of computer code

| | |
|---|---|
| Data collection | Matlab 8.5 (MathWorks) was used to graph and simulate. |
| Data analysis | GraphPad Prism (Version 7) was used to create graphs and conduct statistical analyses. |

For manuscripts utilizing custom algorithms or software that are central to the research but not yet described in published literature, software must be made available to editors and reviewers. We strongly encourage code deposition in a community repository (e.g. GitHub). See the Nature Research guidelines for submitting code & software for further information.

## Data

Policy information about availability of data

All manuscripts must include a data availability statement. This statement should provide the following information, where applicable:
- Accession codes, unique identifiers, or web links for publicly available datasets
- A list of figures that have associated raw data
- A description of any restrictions on data availability

All relevant data are available within the article and Supplementary Information, and from the corresponding author upon reasonable request.



April 2020

# Field-specific reporting

Please select the one below that is the best fit for your research. If you are not sure, read the appropriate sections before making your selection.

☒ Life sciences ☐ Behavioural & social sciences ☐ Ecological, evolutionary & environmental sciences

For a reference copy of the document with all sections, see nature.com/documents/nr-reporting-summary-flat.pdf

# Life sciences study design

All studies must disclose on these points even when the disclosure is negative.

| | |
|---|---|
| Sample size | Sample numbers were selected based on power analyses in most cases. Statistical analyses and number of replicates were determined for each assay and are mentioned separately for each figure. |
| Data exclusions | No data excluded |
| Replication | At least 3 biological replicates assured the reproducibility |
| Randomization | No specific randomization was needed |
| Blinding | In most of the assays, analyses were done blindly |

# Reporting for specific materials, systems and methods

We require information from authors about some types of materials, experimental systems and methods used in many studies. Here, indicate whether each material, system or method listed is relevant to your study. If you are not sure if a list item applies to your research, read the appropriate section before selecting a response.

## Materials & experimental systems

| n/a | Involved in the study | |
|---|---|---|
| ☐ | ☒ | Antibodies |
| ☐ | ☒ | Eukaryotic cell lines |
| ☒ | ☐ | Palaeontology and archaeology |
| ☐ | ☒ | Animals and other organisms |
| ☒ | ☐ | Human research participants |
| ☐ | ☐ | Clinical data |
| ☒ | ☐ | Dual use research of concern |

## Methods

| n/a | Involved in the study | |
|---|---|---|
| ☒ | ☐ | ChIP-seq |
| ☐ | ☒ | Flow cytometry |
| ☒ | ☐ | MRI-based neuroimaging |

## Antibodies

| | |
|---|---|
| Antibodies used | CD3 (1:500 dilution, Biolegend Cat#: 100203), CD11b (1:200 dilution, Biolegend Cat#: 101211), I-A/I-E (1:200 dilution, Biolegend Cat#: 107628) CD19 (1:200 dilution, Biolegend Cat#: 115507) and CD206 (1:200 dilution, Biolegend, Cat#: 141711); CD4 (BioLegend, CAT#: 100512; clone RM4-5), and CD8 (BioLegend, CAT#: 100709; clone 53- 6.7), anti-Armenian hamster IgG (30μg/mL, Jackson Immuno research, CAT#:127-005-099) with CD3 (0.5 μg/mL, Tonbo, CAT#: 70-0031) and CD28 (1 μg/mL, Tonbo, CAT#: 70-0281), αSMA (1:500 dilution, Biolegend Cat#: MMS-466S) and CD68 (1:200 dilution, Biolegend Lot#: B229996), CD3 (1:500 dilution, Biolegend Cat#: 100203), CD11b (1:200 dilution, Biolegend Cat#: 101211), I-A/I-E (1:200 dilution, Biolegend Cat#: 107628) and CD206 (1:200 dilution, Biolegend, Cat#: 141711). |
| Validation | We either used the validated antibodies based on manufacturer protocols or verified in pilot tests using isotype controls. |

## Eukaryotic cell lines

Policy information about cell lines

| | |
|---|---|
| Cell line source(s) | RAW 264.7; THP-1; Human PBMCs |
| Authentication | Immortalized cell lines were authenticated by the manufacturer |
| Mycoplasma contamination | Cells were not tested for Mycoplasma contamination |
| Commonly misidentified lines (See ICLAC register) | NA |

## Animals and other organisms

Policy information about studies involving animals; ARRIVE guidelines recommended for reporting animal research

| | |
|---|---|
| Laboratory animals | Male C57/BL6 mice and Male SD rats |
| Wild animals | Study did not involve wild animals |
| Field-collected samples | Study did not involve field collected samples |
| Ethics oversight | All animal procedures were performed under approved University of California Irvine, Institutional Animal Care and Use Committee (Protocol #: AUP-17-241), in accordance with the guidelines of the National Institutes of Health. |

Note that full information on the approval of the study protocol must also be provided in the manuscript.

## Clinical data

Policy information about clinical studies

All manuscripts should comply with the ICMJE guidelines for publication of clinical research and a completed CONSORT checklist must be included with all submissions.

| | |
|---|---|
| Clinical trial registration | NA |
| Study protocol | NA |
| Data collection | NA |
| Outcomes | NA |

## Flow Cytometry

### Plots

Confirm that:

☒ The axis labels state the marker and fluorochrome used (e.g. CD4-FITC).

☒ The axis scales are clearly visible. Include numbers along axes only for bottom left plot of group (a 'group' is an analysis of identical markers).

☒ All plots are contour plots with outliers or pseudocolor plots.

☒ A numerical value for number of cells or percentage (with statistics) is provided.

### Methodology

| | |
|---|---|
| Sample preparation | Cells were isolated from tissues using digestion or from PBMCs isolated from human blood and/or umbilical cord |
| Instrument | Novocyte 3000 |
| Software | FlowJo v10 was used |
| Cell population abundance | At least 10,000 cells were present per gating |
| Gating strategy | FSC/SSC was used for cells. Double discrimination was used by Height/Area gating of FSC. Dead cells were eliminated from further analyzes using 7AAD- marker. |

☒ Tick this box to confirm that a figure exemplifying the gating strategy is provided in the Supplementary Information.
